# Systemic Chemotherapy in Penile Squamous Cell Carcinoma: Mechanisms, Clinical Applications, and Evidence-Based Regimens

**DOI:** 10.3390/cancers18010046

**Published:** 2025-12-23

**Authors:** Michalina Grudzińska, Mateusz Czajkowski, Maciej Dolny, Marcin Matuszewski, Piotr Mieczysław Wierzbicki, Agnieszka Rybarczyk, Oliver Walther Hakenberg

**Affiliations:** 1 Student Scientific Circle, Department of Urology, Faculty of Medicine, Medical University of Gdańsk, Marii Skłodowskiej-Curie 3a, 80-210 Gdańsk, Poland; 2 Department of Urology, Medical University of Gdańsk, Mariana Smoluchowskiego 17 Street, 80-214 Gdańsk, Poland; mateusz.czajkowski@gumed.edu.pl (M.C.); maciejdolny@gumed.edu.pl (M.D.); marcin.matuszewski@gumed.edu.pl (M.M.); 3 Penile Disease Center, University Clinical Center of Gdańsk, 80-952 Gdańsk, Poland; 4 Department of Histology, Medical University of Gdańsk, Dębinki, 80-211 Gdańsk, Poland; pwierzb@gumed.edu.pl (P.M.W.); agnieszka.rybarczyk@gumed.edu.pl (A.R.); 5 Department of Urology, Jena University Hospital, 07747 Jena, Germany; oliver.hakenberg@med.uni-jena.de

**Keywords:** penile cancer, squamous cell carcinoma, chemotherapy, cisplatin, paclitaxel, neoadjuvant therapy, adjuvant therapy, metastatic disease, guidelines

## Abstract

**Background/Objectives:** Penile squamous cell carcinoma (PSCC) is rare but aggressive. Systemic chemotherapy plays a crucial role in the management of node-positive or metastatic cases; however, the supporting evidence predominantly originates from small, non-randomized studies. This review provides a narrative analysis of the cytotoxic classes and regimens employed in PSCC and compares major clinical guidelines to facilitate informed decision-making in practice. **Methods:** English-language reports were identified in PubMed/Scopus/Google Scholar without date limits. Selection prioritized objective response, survival and toxicity outcomes, and guidance statements across neoadjuvant, adjuvant, and palliative settings. **Results:** Bleomycin-containing triplet regimens demonstrated efficacy but were associated with unacceptable pulmonary toxicity, leading to their discontinuation in clinical recommendations. Currently, cisplatin/taxane-based combinations remain fundamental in treatment protocols. The paclitaxel–ifosfamide–cisplatin (TIP) regimen achieves approximately 40–50% objective responses in phase II studies and may enable curative surgery, while taxane–cisplatin–5-fluorouracil (TPF) shows comparable efficacy with higher toxicity. For less fit patients, cisplatin–5-fluorouracil (PF) or carboplatin–taxane doublets are pragmatic alternatives. Single-agent taxanes or vinflunine offer modest second-line benefits. Although EAU–ASCO 2023, ESMO–EURACAN 2024, and NCCN v2.2025 are broadly in consensus, recommendations differ regarding eligibility thresholds and regimen preferences. Overall, the quality of the evidence remains low. **Conclusions:** TIP remains the reference neoadjuvant option for chemotherapy-fit patients with bulky nodal disease; doublets are reasonable when cisplatin fitness is limited; and bleomycin should be avoided. Harmonized eligibility criteria, biomarker-enriched studies, and coordinated multicenter trials are needed to improve outcomes in this rare malignancy.

## Simple Summary

Penile squamous cell carcinoma (PSCC) is a rare but aggressive malignancy. In its advanced stages, systemic chemotherapy plays a pivotal role; however, the evidence remains limited, and treatment strategies are frequently extrapolated from other cancer types. This review provides a concise and comprehensive overview of the cytotoxic agents used in PSCC, outlining their mechanisms of action, historical development, and current clinical applications. Moreover, major international guidelines are compared to highlight the similarities and differences relevant to daily practice. This review seeks to assist clinicians and researchers in understanding the dynamic landscape of chemotherapeutic options for the treatment of penile cancer.

## 1. Introduction

Penile squamous cell carcinoma (PSCC) remains a rare malignancy in Europe and the United States (<1% of all cancers). In 2020, the global age-standardized incidence and mortality were 0.8 and 0.29 per 100,000 men, respectively (36,068 cases; 13,211 deaths) [1,2]. The global burden of this condition shows significant variation, with incidence rates notably higher in certain regions of Africa, Asia, and South America, where PSCC may account for approximately 10% of all cancer diagnoses [3]. In contrast, it remains relatively rare in highly developed regions. In Europe, both incidence and mortality are strongly age-dependent: >98% of cases and deaths occur in men aged ≥40 years, peaking at 60–74 years. Incidence has increased in several European nations, and mortality has risen in some—including Poland—underscoring the need for coordinated, centralized care and multidisciplinary decision-making [4]. Survival rates are dependent upon the stage of the disease: five-year overall survival approaches ~90% for organ-confined disease, declines to ~80% with limited unilateral nodal involvement, falls to ~10–20% with bilateral or pelvic nodal metastases, and is <10% in the presence of extranodal extension or distant metastases [5]. Primary management is surgical, ranging from organ-preserving wide local excision and glansectomy to partial penectomy with reconstruction and, when required, total penectomy [3,6]. In cases of node-positive disease, regional lymphadenectomy, including inguinal lymph node dissection (ILND) and/or pelvic lymph node dissection (PLND), is a fundamental treatment component and is frequently integrated with systemic therapy [7]. Within this multimodal approach, chemotherapy fulfills three primary functions: neoadjuvant chemotherapy (NAC) aims to downstage bulky, fixed, or otherwise high-risk nodal disease to facilitate definitive surgical intervention; adjuvant chemotherapy (AC) is employed for adverse pathological features post lymphadenectomy, such as pN2–pN3 and extranodal extension; and palliative therapy is utilized for unresectable or metastatic disease [8,9,10]. Current guidelines (EAU–ASCO 2023, ESMO–EURACAN 2024, NCCN v2.2025) [8,9,10] consistently recommend cisplatin- and taxane-based combinations as the cornerstone of systemic chemotherapy: triplet regimens such as TIP (paclitaxel–ifosfamide–cisplatin) or TPF (docetaxel–cisplatin–5-FU) demonstrate objective response rates of approximately 40–50% in phase II cohorts. For patients with reduced fitness, PF (cisplatin–5-FU) or carboplatin–paclitaxel are considered reasonable alternatives, while regimens containing bleomycin are avoided due to the risk of pulmonary toxicity [8,9,10]. High-quality randomized data remain scarce, second-line benefit is modest (median overall survival often ≤6 months), and criteria differ across guidelines.

This narrative review synthesizes the mechanisms, indications, efficacy, and toxicity of key cytotoxic agents; it compares international recommendations, highlighting areas of consensus and divergence, and outlines current evidence gaps and trial priorities. Furthermore, we provide a side-by-side summary of indications and preferred regimens to support practical decision-making in centralized PSCC pathways.

## 2. Materials and Methods

We performed a narrative review focused on systemic cytotoxic therapy in PSCC. Sources included prospective and retrospective clinical studies and international guidelines (EAU–ASCO, ESMO–EURACAN, NCCN). Abstracts and other forms of unpublished preliminary reports were excluded from the review. Ongoing clinical trials were included into the analysis when properly documented by an official trial registration number. English-language records were searched in PubMed/Scopus/Google Scholar. Data were collected between March 2025 and November 2025. Studies published up to November 2025 were eligible, with no lower limit on publication year. Study selection prioritized reports with objective response, survival and toxicity data, and guideline statements on indications/dosing. Studies including treatment with systemic chemotherapy alone, without concurrent non-surgical therapies such as immunotherapy and radiotherapy, were prioritized, although if such studies were identified, this was appropriately noted in the Results section. This review is not a systematic review and does not follow PRISMA [11].

## 3. Results

### 3.1. Historical Evolution of Chemotherapy Regimens

#### 3.1.1. Era Before Systemic Therapy

Before systemic therapy, outcomes were poor: radical surgery (e.g., hemipelvectomy) or radiotherapy provided, at best, variable local control and had little impact on node-positive or advanced disease [12,13,14].

#### 3.1.2. Single-Agent Attempts (1960–1980s)

Early systemic chemotherapy trials were conducted from the 1960s to the 1980s using single agents such as bleomycin, cisplatin, or methotrexate. In 1969, Ichikawa et al. were the first to describe the use of bleomycin in the treatment of PSCC, reporting an objective response rate of 75% among eight patients, including one complete response [15]. All further trials demonstrated moderate efficacy, with any meaningful responses largely restricted to isolated case reports [16,17,18,19,20,21,22].

#### 3.1.3. Early Doublets: Limited Efficacy

Initial two-drug combinations failed to deliver consistent benefits. Williams et al. (1974) observed no responses in three patients treated with vincristine plus bleomycin; similar negative results were seen with bleomycin alone or combined with actinomycin D or vinblastine in advanced stages [23,24]. These disappointing data set the stage for triplet strategies that pursued higher activity at the cost of greater toxicity.

#### 3.1.4. Bleomycin-Containing Triplets (Vincristine–Bleomycin–Methotrexate (VBM)/Dexeus): Activity Offset by Toxicity

The first described triple-agent approach in PSCC was VBM. In 1988, Pizzocaro and Piva reported adjuvant/neoadjuvant VBM treatment with good responses but notable bleomycin-related pulmonary toxicity; thus, VBM showed proof-of-concept efficacy but proved too toxic to be used as standard therapy [25]. Further, multiagent platinum regimens came into focus, first incorporating the cisplatin–bleomycin–methotrexate triplet, in what became known as the “Dexeus regimen” described by Dexeus et al. in 1991 [26]. They observed 10 responses among 14 patients (12 with penile primary tumors), although bleomycin-related toxicity was observed in 5 cases [26]. In subsequent trials, the most comprehensive of which was conducted by Haas et al. in 1999 under the Southwest Oncology Group (SWOG), only 13 responses were observed among 40 patients [27]. Additionally, there were five treatment-related fatalities, which led to the discontinuation of bleomycin-containing regimens in contemporary practice due to their unacceptable toxicity [27].

#### 3.1.5. PF Doublets (Cisplatin + 5-FU): Pragmatic Activity

Concurrently, reports on PF (cisplatin-5-fluorouracil) began to emerge, initially documented by Hussein et al. in 1990 as a case series involving five men with advanced PSCC and one with urethral SCC [28]. Tumor regression was achieved in all six patients, with two subsequently rendered disease-free through surgical intervention [28]. Another small-scale trial demonstrated the limited efficacy of PF, with two out of eight patients with advanced disease showing partial responses [29].

#### 3.1.6. Taxanes Enter the Field: TP Doublets (Paclitaxel + Platinum) and Monotherapy

The early 2000s marked the introduction of taxane-based chemotherapy in PSCC. The first report by Joerger et al. (2004) described a single patient with a nodal metastasis treated with a paclitaxel–carboplatin doublet, resulting in marked tumor regression [30]. Subsequently, Bermejo et al. (2007) applied the same combination in a neoadjuvant setting for two patients, both of whom achieved no evidence of disease following lymphadenectomy [31]. Researchers also investigated both paclitaxel–carboplatin and paclitaxel–cisplatin regimens in studies of adjuvant (Noronha et al. 2012 [32]) and palliative therapy (Patil et al. 2014 [33]), showing positive outcomes with both doublets. Taxane monotherapy was also explored; Di Lorenzo et al. (2011) reported partial responses in 20% of pretreated metastatic penile cancer patients [34].

#### 3.1.7. Modern Triplets: TIP Versus TPF

The modern era began with TIP (paclitaxel–ifosfamide–cisplatin). In Bermejo et al. (2007), neoadjuvant TIP produced responses in four out of five selected metastatic patients with a 5-year survival of 40% [31]. In the phase II study by Pagliaro et al. (2010) (*n* = 30), neoadjuvant TIP achieved a 50% ORR (including 3 CR) and median OS of 17.1 months [35]. By contrast, Sitompul et al. (2019) reported a median OS of 6 months, noting that only 10/17 patients completed all four cycles, underscoring the impact of selection and treatment compliance [36]. In parallel, TPF (taxane–platinum–5-FU) was evaluated. Pizzocaro et al. (2009) (paclitaxel–cisplatin–5-FU) reported two instances of pathological CR among six patients and three additional responders [37]. A pilot study by Salvioni (2011), later expanded by Nicolai (2016), confirmed activity in node-positive disease (43% response neoadjuvantly) after switching from paclitaxel to docetaxel in 2007, while the adjuvant role remained controversial [38,39]. Two further docetaxel–cisplatin–5-FU series showed 38.5% responses (CRUK/09/001, Nicholson 2013; Zhang 2015), with frequent grade-3–4 toxicity in the former [40,41]. A 2024 case report described a paclitaxel–5-FU variant with carboplatin substituted for cisplatin due to renal failure, yielding a favorable outcome.

#### 3.1.8. Second-Line and Orphan Options

In 2022, the UK phase II VinCaP trial was the first to evaluate vinflunine monotherapy in advanced PSCC, achieving an objective response and stable disease in 45.5% of the 22 patients, thereby meeting the primary endpoint. However, 68% of patients experienced at least one grade-3 toxicity [42]. While the TIP regimen remains the standard first-line/neoadjuvant treatment for fit patients [35,36,43], a recent comparative analysis by Dhasthakeer et al. (2025) indicates that paclitaxel–carboplatin may provide the most favorable balance of efficacy and tolerability among commonly used doublet/triplet regimens [44].

#### 3.1.9. Summary for Practice

**Figure 1:**
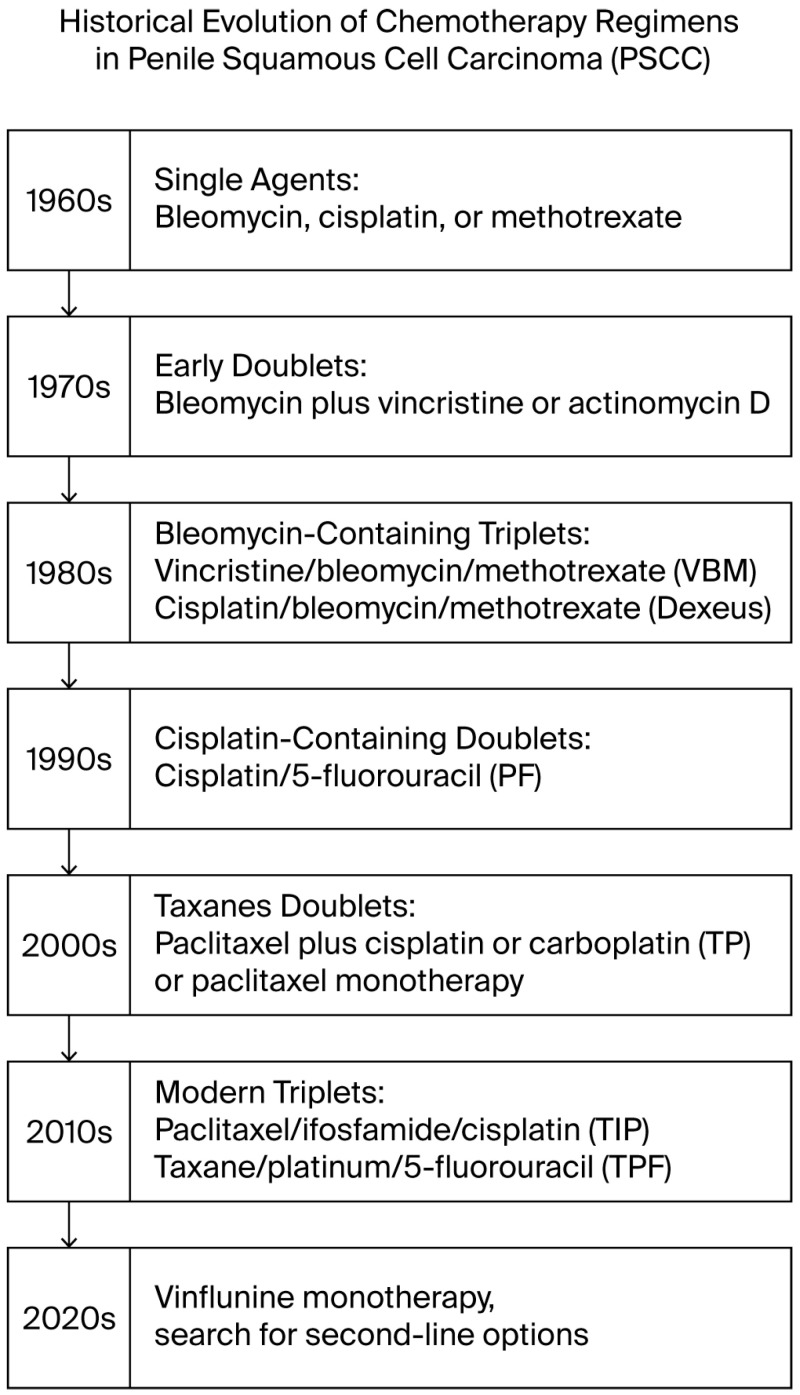
Timeline of the historical evolution of chemotherapy regimens in penile squamous cell carcinoma (PSCC).

In summary, systemic regimens for penile SCC evolved from bleomycin-based triplets (VBM/Dexeus) with high toxicity and variable efficacy and platinum/5-FU doublets yielding modest responses to modern taxane-containing combinations (TIP, TPF), with roughly half of patients responding. These developments have marginally extended survival in metastatic disease (median OS rising from <6 months historically to >1 year with TIP). Nonetheless, complete remission remains rare, and ongoing trials seek to improve these outcomes [17,45]. An overview timeline outlining the chronological evolution of key systemic chemotherapy milestones is provided in Figure 1. Key regimen data are summarized in Table 1. Expanded information, including treatment regimen details, completion of planned cycles, indications, toxicity assessed according to Common Terminology Criteria for Adverse Events (CTCAE) [46], concurrent non-surgical therapies, study design, and level of evidence based on the Oxford Centre for Evidence-Based Medicine (OCEBM) criteria [47] is provided in Supplementary Table S1. In addition, evidence summaries of chemotherapy regimens in PSCC for bleomycin-containing, methotrexate monotherapy, and other historical regimens (1965–2000) are presented in Supplementary Table S2, while summaries of emerging, second-line, and other contemporary regimens are provided in Supplementary Table S3.

### 3.2. Mechanistic Drug Classes

#### 3.2.1. Alkylating Agents—Metal Salts

##### Cisplatin

In PSCC, cisplatin is the preferred platinum agent within the PF, TP, and TIP/TPF regimens for eligible patients. The selection is influenced by nephro-, oto-, and neurotoxicity, as determined by Eastern Cooperative Oncology Group Performance Status (ECOG PS), glomerular filtration rate (GFR), and neuropathy assessments [8,9,10,57,58,59,60,61,62,63,64,65,66,67,68,69,70,71,72].

##### Carboplatin

Carboplatin serves as an alternative to cisplatin and is effectively combined with paclitaxel in patients with reduced fitness [70,71,73,74,75,76].

#### 3.2.2. Alkylating Agents—Mustard Gas Derivatives

##### Ifosfamide

In PSCC, ifosfamide is included in TIP regimens. Dose-limiting toxicities are hemorrhagic cystitis (prevented by Mesna), myelosuppression, and neurotoxicity [77,78,79,80,81,82,83,84,85].

#### 3.2.3. Antimetabolites—Pyrimidine Antagonists

##### 5-Fluorouracil

This compound is employed in the treatment of PSCC in conjunction with cisplatin as part of TPF/PF regimens. Notable toxicities associated with this treatment include gastrointestinal mucositis and myelosuppression [86,87,88,89,90,91,92,93].

##### Gemcitabine

In PSCC, gemcitabine was used in one phase II trial and in retrospective studies with little efficacy, either as monotherapy or in combination with cisplatin. However, it is not currently included in treatment guidelines. Its primary toxicity is myelosuppression [94,95,96,97,98,99,100,101,102,103,104,105,106].

#### 3.2.4. Antimetabolites—Folic Acid Antagonists

##### Methotrexate

Although it was historically included in cisplatin–methotrexate–bleomycin (BMP) regimens, methotrexate currently has no place in PSCC guidelines. However, if considered in regimens excluding bleomycin, the toxicities associated with methotrexate, such as myelosuppression, gastrointestinal toxicity, and neurotoxicity, can be mitigated by the administration of leucovorin [107,108,109,110,111,112,113,114,115,116,117,118,119].

#### 3.2.5. Antitumor Antibiotics—Anthracyclines

##### Doxorubicin (Adriamycin)

Doxorubicin is not recommended for PSCC, as its application is limited by cumulative cardiotoxicity [120,121,122,123,124,125,126,127,128,129].

##### Epirubicin

This doxorubicin analog exhibits comparable antineoplastic efficacy with reduced cardiotoxicity. However, its application in PSCC is controversial and is not recommended [120,130,131,132,133,134,135,136,137].

#### 3.2.6. Antitumor Antibiotics—Miscellaneous

##### Bleomycin

Current guidelines explicitly advocate against the use of bleomycin in PSCC due to a high risk of pulmonary fibrosis [8,10,138,139,140,141,142,143,144,145,146,147].

##### Mitomycin C

In guidelines, the use of mitomycin C is limited to a recommendation for palliative chemoradiotherapy (in combination with 5-fluorouracil or capecitabine, based on evidence extrapolated from other types of SCC). The main toxicities include cumulative myelosuppression and pulmonary toxicity [10,148,149,150,151].

#### 3.2.7. Plant Alkaloids—Vinca Alkaloids

##### Vincristine

In PSCC, vincristine was used in combination with bleomycin-containing regimens, which are no longer in use; vincristine-induced peripheral neuropathy is a major toxicity [19,23,25,52,152,153,154,155,156,157,158].

##### Vinblastine

In PSCC, vinblastine only appears in combination regimens that are not guideline-recommended; its notable dose-limiting toxicity is myelosuppression [119,120,157,159,160,161,162,163].

##### Vinflunine

In the context of monotherapy for the palliative treatment of PSCC, vinflunine is recommended for patients who are unsuitable for cisplatin. This recommendation is derived from a single phase II trial. Among the reported toxicities, neutropenia is common but generally manageable [8,42,159,164,165,166,167,168,169,170,171,172].

#### 3.2.8. Plant Alkaloids—Taxanes

##### Paclitaxel

Within PSCC treatment regimens, paclitaxel is incorporated as part of the first-line TIP therapy or TP for patients with reduced fitness. For those unfit for cisplatin due to decreased GFR but with an acceptable ECOG PS, it is administered either as a paclitaxel/carboplatin combination or as monotherapy. Patients with pre-existing neuropathy are typically excluded from treatment, as the drug may induce dose-limiting peripheral neuropathy [8,9,10,34,173,174,175,176,177,178,179,180,181].

##### Docetaxel

Docetaxel is utilized in TPF and taxane–platinum regimens or as a single agent, similar to paclitaxel, in PSCC, following the same eligibility criteria. The primary toxicities associated with its use are neutropenia and peripheral neuropathy [8,9,10,181,182,183,184,185,186,187,188,189,190].

#### 3.2.9. Rarely Used Agents

Several agents have been explored sporadically and mentioned in PSCC literature without an established role in current guidelines, including pirarubicin, an analog of doxorubicin; peplomycin, a bleomycin derivative; actinomycin D, a DNA-intercalating antibiotic; vinorelbine, a vinca alkaloid; etoposide, a topoisomerase II inhibitor; cabazitaxel, a second-generation taxane; oxaliplatin, a third-generation platinum derivative; and irinotecan, a topoisomerase I inhibitor, evaluated with cisplatin in the EORTC phase II trial in advanced PSCC but not adopted in guidelines [8,9,10,75,162,181,191,192,193,194,195,196]. However, one of the rarely used agents, capecitabine, an oral prodrug of 5-FU with reduced systemic toxicity, is mentioned in one of the guidelines as a monotherapy option in chemoradiotherapy protocols extrapolated from other SCCs [10,197].

#### 3.2.10. Summary of Mechanistic Drug Classes in PSCC Management

All of the chemotherapeutic agents described above are summarized in Table 2, which presents their mechanisms of action, key clinical notes, and published references supporting their use in PSCC in a compact form. Moreover, a detailed description of individual agents, including their historical development and mechanisms of action across other malignancies, is provided in Supplementary Text S1.

**Figure 2:**
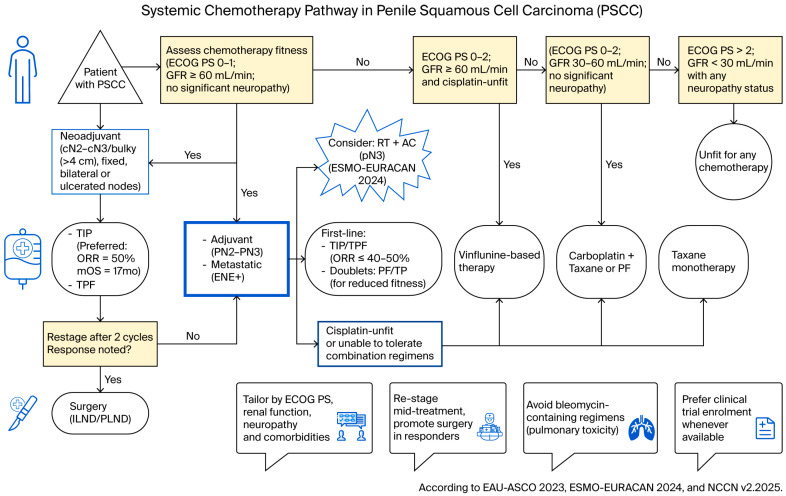
Flowchart of the systemic chemotherapy pathway in penile squamous cell carcinoma (PSCC). AC: adjuvant chemotherapy; ASCO: American Society of Clinical Oncology [8]; cN: clinical nodal status; ECOG PS: Eastern Cooperative Oncology Group Performance Status; EAU: European Association of Urology [8]; ENE+: extranodal extension; ESMO: European Society for Medical Oncology [9]; EURACAN: European Reference Network for Rare Adult Solid Cancers [9]; GFR: glomerular filtration rate; ILND: inguinal lymph node dissection; mOS: median overall survival; NCCN: National Comprehensive Cancer Network [10]; ORR: objective response rate; PF: cisplatin + 5-fluorouracil; PLND: pelvic lymph node dissection; pN: pathological nodal status; PSCC: penile squamous cell carcinoma; RT: radiotherapy; TIP: paclitaxel + ifosfamide + cisplatin; TP: taxane + platinum; TPF: taxane + cisplatin + 5-fluorouracil.

In cisplatin-eligible patients with bulky lymph node involvement, neoadjuvant TIP is preferred to maximize tumor response prior to lymphadenectomy. For those unfit for cisplatin, treatment should be adapted: if partial eligibility allows for reduced dosing, cisplatin combined with 5-FU (PF) may be used; if cisplatin is entirely contraindicated, a carboplatin–taxane regimen such as paclitaxel–carboplatin or taxane monotherapy can be an alternative. Bleomycin-containing regimens should be avoided due to high pulmonary toxicity risk. After the initial chemotherapy cycles, early restaging with repeat imaging is recommended to assess response and identify patients whose nodal disease has been downstaged, as these patients should then undergo surgical lymph node dissection. Ultimately, regimen selection should be individualized to patient factors, considering ECOG PS, renal function (GFR), pre-existing neuropathy, and anticipated toxicity profile. A graphical flowchart (Figure 2) summarizing the clinical pathway in PSCC is presented below.

### 3.3. Clinical Applications of Chemotherapy in PSCC

Systemic chemotherapy in PSCC plays a role in both neoadjuvant and adjuvant settings and, likewise, in palliative care. Its use is guided by disease stage, nodal burden, and patient fitness, as outlined in major international guidelines, including the EAU-ASCO 2023 [8], ESMO-EURACAN 2024 [9], and NCCN 2.2025 [10].

#### 3.3.1. Neoadjuvant Chemotherapy in PSCC

Neoadjuvant chemotherapy (NAC) is recommended in patients with locally advanced or high-risk penile squamous cell carcinoma (PSCC) [215,216], particularly those with inguinal or pelvic nodal involvement (cN2–cN3) [217] prior to inguinal lymph node dissection (ILND) and/or pelvic lymph node dissection (PLND). It is particularly recommended in cases with bulky (>4 cm), fixed, bilateral, or ulcerated nodes [31,35,51,218] and in T4 primary tumors deemed initially unresectable [219].

Cisplatin- and taxane-based triplet regimens constitute the standard of care. The most commonly used are TIP (paclitaxel–ifosfamide–cisplatin) [35] and TPF (docetaxel–cisplatin–5-FU) [39,40,50]. NAC allows for the early treatment of micrometastatic disease, nodal downstaging, and subsequent surgical resection in responders, with response rates approaching 50% and long-term disease-free survival exceeding 30% in phase II studies [35,218].

However, the lack of randomized trials limits the level of evidence, and patient selection remains critical—NAC should be reserved for chemotherapy-fit patients (ECOG PS 0–1, intact renal function). Triplet regimens should be avoided in patients with baseline neuropathy or impaired glomerular filtration.

#### 3.3.2. Adjuvant Chemotherapy in PSCC

Adjuvant chemotherapy is considered in PSCC patients with adverse pathological features after lymph node dissection, including multiple positive nodes, pelvic nodal involvement, extranodal extension, or metastases larger than 30 mm (pN2–pN3) [107,220,221,222].

The same cisplatin-based regimens as in NAC (TIP [221,223] or TPF [39,51,223]) are recommended, with PF (5-FU–cisplatin) as an alternative [29,54,55,221]. Evidence supporting the adjuvant use is limited to retrospective analyses [107,220,221,223]; therefore, guidelines emphasize shared decision-making and individualized patient counseling.

Notably, ESMO-EURACAN 2024 highlights the potential benefit of combining adjuvant chemoradiotherapy in pN3 disease, with improved cause-specific survival (29% vs. 16%) compared with chemotherapy alone [224] and higher 2-year OS compared with single-modality or no adjuvant treatment (75% versus 67% and 28%, respectively) [220].

#### 3.3.3. Palliative Chemotherapy in PSCC

Palliative systemic chemotherapy is offered to patients with distant metastases; recurrent or locally advanced disease is not amenable to curative surgery [55,215,216].

Platinum-based combinations remain the cornerstone of treatment. TIP and TPF achieve objective response rates of 38–50%, with a median OS of 7–14 months [35,40,41]. For cisplatin-unfit patients, PF and carboplatin–paclitaxel doublets provide more tolerable alternatives [29,33,55], though with lower activity (ORR 32%, median OS 8 months) [55].

Single-agent taxanes or vinflunine can offer modest benefits in subsequent lines, with ORRs of 20–27% in prospective phase II trials [34,42]. Bleomycin-containing regimens are contraindicated due to their high risk of fatal pulmonary toxicity [27]. Second-line systemic therapy yields limited outcomes (median OS ≤ 6 months [200,204]); therefore, clinical trial enrolment or best supportive care are recommended whenever possible.

### 3.4. Comparison of Guidelines

A comparative overview of the current EAU–ASCO 2023, ESMO–EURACAN 2024, and NCCN 2.2025 guidelines shows broad consensus regarding the indications and general strategy of systemic chemotherapy in PSCC, alongside notable differences in regimen selection, patient fitness criteria, and integration with radiotherapy [8,9,10].

#### 3.4.1. Similarities in Guidelines

Across the EAU–ASCO 2023, ESMO–EURACAN 2024, and NCCN v2.2025 guidelines, there is broad consensus regarding the clinical use of systemic chemotherapy in PSCC. All three emphasize the central role of cisplatin- and taxane-based multi-agent regimens in the neoadjuvant, adjuvant, and palliative settings, while uniformly discouraging the use of bleomycin due to its pulmonary toxicity and lack of benefit in contemporary studies [8,9,10].

Neoadjuvant chemotherapy (NAC) is recommended for chemotherapy-fit patients with bulky, fixed, bilateral, or pelvic nodal disease (cN2–cN3) and for locally advanced T4 tumors initially considered unresectable. The goal is to achieve downstaging, eradicate micrometastatic disease, and facilitate subsequent curative lymphadenectomy. The TIP regimen is the most consistently endorsed option, achieving an ORR of approximately 40–50% and pCR in up to 10% of patients in prospective phase II trials. TPF is accepted as an alternative, though it is associated with higher hematologic toxicity [8,9,10].

Adjuvant chemotherapy (AC) is considered in patients with pN2–pN3 disease, extranodal extension (ENE), or pelvic lymph node involvement following lymphadenectomy, particularly when NAC was not previously administered. In this setting, the same cisplatin-based regimens (TIP or TPF) are used, with PF (cisplatin–5-FU) as a pragmatic option for less fit individuals. Evidence remains largely retrospective, and all guidelines stress individualized decision-making based on pathological stage, renal function, and performance status [8,9,10].

In the palliative setting, platinum-based regimens continue to represent the backbone of systemic therapy. Both TIP and TPF demonstrate consistent activity, with an ORR of 38–50% and median OS ranging from 7 to 14 months. For cisplatin-unfit or frail patients, doublet regimens such as PF or carboplatin–paclitaxel are recommended, offering more favorable tolerability at the expense of efficacy. Single-agent taxanes or vinflunine are reasonable later-line options, providing modest benefit (ORR 20–27%) and manageable toxicity [8,9,10].

All guidelines agree that second-line systemic therapy provides limited survival benefit (median OS ≤ 6 months) and strongly encourage clinical trial enrolment for patients with advanced or relapsed PSCC. Collectively, these recommendations support a unified therapeutic framework centered on cisplatin-based chemotherapy for fit patients, carboplatin- or fluorouracil-based alternatives for unfit patients, and the avoidance of bleomycin-containing regimens [8,9,10].

#### 3.4.2. Differences in Guidelines

Despite shared foundations, the EAU–ASCO 2023, ESMO–EURACAN 2024, and NCCN v2.2025 guidelines differ in their specificity, therapeutic scope, and toxicity management for systemic chemotherapy in PSCC. The NCCN guidelines provide the most prescriptive framework, defining explicit staging and size thresholds for neoadjuvant therapy (e.g., ≥4 cm inguinal nodes, TX N2–3 M0). In contrast, EAU–ASCO allows the broadest therapeutic flexibility, recommending both doublet and triplet cisplatin-based regimens in neoadjuvant and adjuvant settings. ESMO–EURACAN adopts a more conservative approach, limiting both neoadjuvant and adjuvant chemotherapy to triplet regimens only.

The NCCN restricts neoadjuvant therapy exclusively to TIP (paclitaxel–ifosfamide–cisplatin), explicitly excluding PF (cisplatin–5-FU) in this context, although PF is accepted as an alternative in the adjuvant setting. ESMO–EURACAN uniquely emphasizes the survival advantage of adjuvant chemoradiotherapy in pN3 disease with improved cause-specific survival compared with chemotherapy alone. Conversely, EAU–ASCO highlights the difficulty of optimal patient selection due to the absence of randomized data and therefore grades adjuvant chemotherapy recommendations as weak.

For palliative first-line therapy, only EAU–ASCO explicitly specifies regimens for platinum-ineligible patients, recommending vinflunine or taxanes as reasonable alternatives. In contrast, NCCN uniquely details second-line palliative options, including paclitaxel monotherapy, and combined chemo/radiotherapy protocols (e.g., cisplatin alone, PF, mitomycin C + 5-FU, or capecitabine).

Toxicity management also varies between the guidelines. EAU–ASCO specifically cautions against the use of TIP or TPF in patients with baseline neuropathy or impaired renal function, while ESMO–EURACAN prioritizes tailoring systemic therapy according to ECOG performance status, putting the focus on patient fitness. Collectively, these differences highlight the persistent gaps in high-level evidence and the urgent need for prospective multicenter research to harmonize systemic treatment algorithms and optimize patient selection in PSCC management.

#### 3.4.3. Evidence-Based Overview of the Clinical Application of Guidelines

A comprehensive comparative summary of guideline recommendations is presented in Table 3.

### 3.5. Evidence Summary of Chemotherapy Regimens in PSCC

We provide a comprehensive summary of therapeutic regimens for PSCC identified in the available literature, as outlined in the Methods section, encompassing both monotherapy and combination approaches. The analysis includes detailed information on the agents used, dosing schedules, treatment indications, study design, clinical outcomes, and reported toxicities, as well as the historical context of each regimen. The potential impact of concurrent non-surgical modalities was also considered, and each study was evaluated according to the Oxford Centre for Evidence-Based Medicine (OCEBM) 2011 Levels of Evidence [47]. Furthermore, we assessed whether the identified regimens are currently considered in the EAU–ASCO 2023 Collaborative Guidelines. A detailed overview of the reviewed regimens is presented in Table 1 and Supplementary Tables S1–S3.

Overall, the quality of the available evidence remains low. Most studies are retrospective, lack randomization and control groups, and include small, heterogeneous patient cohorts with variable treatment protocols and incomplete or inconsistently reported outcomes. According to the OCEBM criteria [47], the majority of data correspond to Level IV evidence. High-quality prospective or phase III clinical trials are largely unavailable, and follow-up durations are often insufficient to draw firm conclusions regarding long-term efficacy or safety. These limitations should be acknowledged when interpreting the summarized data and applying them to clinical practice.

## 4. Discussion

### 4.1. Mechanistic Rationale for Cytotoxic Combination Therapy in PSCC

Systemic chemotherapeutic agents exploit fundamental vulnerabilities in tumor biology, and in PSCC, they target key pathways of DNA replication and cell-cycle control to suppress tumor growth. The most commonly used platinum-based alkylating agents, such as cisplatin and carboplatin, form DNA crosslinks that block DNA replication and transcription, ultimately triggering apoptosis [57,70].

Another alkylating agent relevant in PSCC is ifosfamide, which covalently binds DNA to form intra- and inter-strand crosslinks and generates reactive oxygen species, causing irreparable DNA damage and cell death. This mechanism confers higher cytotoxic potential than that of platinum agents, as the resulting crosslinks are more resistant to cellular repair [79,85].

In contrast to alkylating agents, antimetabolites such as 5-FU do not directly damage DNA, but instead disrupt the metabolic pathways required for nucleotide synthesis, inhibiting thymidylate synthase (TYMS) and blocking the production of thymidine [86,93]. In addition, gemcitabine causes chain termination and inhibits ribonucleotide reductase, depleting the pool of deoxynucleotides [100,105]. These dual actions block DNA elongation more directly than 5-FU’s upstream blockade of nucleotide synthesis. Another important antimetabolite in PSCC treatment is MTX, which blocks DHFR, depleting reduced folate cofactors. This upstream inhibition suppresses the production of essential DNA precursors [110,114].

Moving from antimetabolites to antitumor antibiotics, the next key group is anthracyclines, represented by doxorubicin (adriamycin) and epirubicin. Unlike antimetabolites, anthracyclines act directly on DNA, which cause DNA strand breaks and block replication [132]. Among antitumor antibiotics, bleomycin is commonly used for metastatic testicular cancer, but not in PSCC, where patients are older and more vulnerable to pulmonary toxicity [8,144]. Another antibiotic, mitomycin C, has been used sporadically in PSCC [148].

The next class comprises plant alkaloids—specifically the vinca alkaloids (vincristine, vinblastine, vinflunine)—which block mitotic spindle formation, thereby inducing metaphase arrest [157]. Among plant alkaloids, vincristine is noted for greater neurotoxicity [152] and vinblastine for more pronounced myelosuppression [161], while vinflunine, a newer fluorinated analog, offers a somewhat improved toxicity profile while retaining antimitotic activity. It was evaluated in the VinCaP Trial reported by Nicholson et al., with promising results in a palliative setting [42].

Taxanes (paclitaxel and docetaxel), which form the backbone of modern PSCC regimens, stabilize microtubules and prevent depolymerization, resulting in mitotic arrest [176,188]. Docetaxel has greater microtubule binding affinity and longer intracellular retention than paclitaxel, with reduced neurotoxicity and hypersensitivity [185], whereas paclitaxel remains widely used but requires more intensive premedication [35].

Beyond these major classes, several other cytotoxic drugs (pirarubicin, peplomycin, actinomycin D, vinorelbine, capecitabine, irinotecan, etoposide, cabazitaxel, and oxaliplatin) have only been reported in isolated cases or small series [56,119,130,208,209,210,211]. Some have been discontinued, whereas others remain experimental and warrant further study [191,200,204,206,214,225].

Understanding these mechanisms underpins the rationale for combination chemotherapy, which aims to attack PSCC cells through multiple cell-cycle vulnerabilities—for example, by pairing DNA-damaging agents (cisplatin, ifosfamide) with anti-mitotics (taxanes) or nucleotide synthesis inhibitors (5-FU).

### 4.2. Comparison of Regimen Efficacy in PSCC

Clinical experience in PSCC has focused on the regimens summarized in Table 1. The standard neoadjuvant regimen is TIP. In a landmark phase II trial conducted by Pagliaro et al., four cycles of TIP achieved a 50% ORR, including 10% of pCR and significantly improved PFS and OS to a median of 8.1 and 17.1 months, respectively [35]. Moreover, this regimen was also investigated in other series with an ORR over 50% in all of them [31,36,44,48], with one exception in a study by Ma et al., who reported an ORR of 47.5% [43]. Notably, TIP demonstrated manageable toxicity and no treatment-related deaths in all analyzed studies [31,35,36,43,44,48].

In contrast, the TPF regimen showed inferior results in the two key phase II studies reported by Zhang et al. [41] and Nicholson et al. In the CRUK/09/001 trial [40] of docetaxel–PF, they reported a 38.5% ORR and grade-3/4 toxicity in 41% and 67.9% of patients, respectively [40,41]. These researchers concluded that TPF offers no advantage over TIP in the neoadjuvant setting. However, it is still relevant for stage M1 patients [41]. Other small studies have reported higher ORRs but were limited by low levels of evidence [37,38,39,50,51].

In the adjuvant or palliative context, PF remains the most frequently used doublet. Despite the absence of phase II data, retrospective series reported an ORR of 25–57%, albeit in heterogeneous populations [29,53,55,120]. Similarly, TP (taxane/platinum) regimens were studied, but the results were conflicting and based on low levels of evidence [30,31,32,33,44].

Earlier, bleomycin-containing combinations (VBM, Dexeus (BMP)) demonstrated activity (ORR~ 25–72) [26,27,107,198]; however, the pulmonary toxicity associated with bleomycin has proven prohibitive, and such regimens have not been investigated in modern clinical trials.

Therefore, in recent times, the focus has shifted toward identifying palliative or second-line therapies with meaningful activity and an acceptable toxicity profile. For example, cisplatin combined with irinotecan was evaluated in the phase II trial by Theodore et al. [191]; although the ORR was only 28.6% in the neoadjuvant setting, the same combination achieved a slightly higher ORR of 31.6% when introduced for palliative treatment. Another phase II effort, the VinCaP trial, assessed vinflunine monotherapy and reported an ORR of 27.3% in the palliative setting [42], offering a potential second-line option for patients with platinum-resistant disease. Similarly, paclitaxel monotherapy was used by Di Lorenzo et al. [34] as a second-line treatment for metastatic patients, achieving an ORR of 20%. Several other palliative regimens have yielded even more disappointing outcomes. For instance, cisplatin–gemcitabine (Houédé et al. [201], ORR 8%), cabazitaxel (Challapalli et al. [214], no objective responses), and mitomycin C in a second-line setting (Draeger et al. [212], ORR 11%) demonstrated very limited efficacy.

Overall, these data confirm TIP as the benchmark regimen, although this conclusion is limited by the absence of large prospective randomized trials. Second-line options offer only modest benefit, highlighting the need for well-designed multicenter trials in advanced PSCC.

### 4.3. Treatment Selection According to Patient Profile (Cisplatin-Fit/Unfit)

Cisplatin remains the cornerstone of systemic therapy but is limited by dose-dependent nephro- and ototoxicity, peripheral neuropathy, and severe nausea/emesis. Carboplatin is associated with lower renal and auditory toxicity, but greater myelosuppression, and is commonly used when cisplatin is contraindicated [118].

A major emerging problem is acquired cisplatin resistance. Recent preclinical models show that PSCC can become resistant to cisplatin when the tumor loses the PTEN tumor suppressor (especially on a SMAD4/APC-deficient background) [226]. This mechanistic insight highlights why half of PSCC patients may not benefit from first-line platinum therapy [35] and underscores the need for alternatives or other combinations (such as immune-based therapies) in advanced disease.

### 4.4. Patient Selection Criteria for Chemotherapy in PSCC

Patient selection for systemic chemotherapy in PSCC is crucial and recommendations are provided in the EAU-ASCO Collaborative Guidelines 2023 [8], which emphasize the reservation of this treatment method for patients who are sufficiently fit and have high-risk disease. Specifically, candidates should have good functional status (e.g., ECOG PS 0-1) and good renal function (normal creatinine clearance or GFR ≥ 60 mL/min). Other comorbidities should be carefully considered as well. The extent of nodal involvement is also an important determining factor: it is recommended that patients with bulky or bilateral inguinal nodes (cN2) or pelvic nodal metastases (cN3) receive neoadjuvant (NAC) cisplatin- and taxane-based chemotherapy before surgery. After definitive surgery, adjuvant chemotherapy may be offered for pathologic N3 disease (extensive nodal metastases) if NAC was not given.

In the metastatic/palliative setting, first-line platinum-based chemotherapy is standard: regimens include multiagent combinations (TIP or TPF) or doublets such as PF or paclitaxel + carboplatin. Again, renal function and comorbidities must be considered. Alternatives are carboplatin-containing doublets or single agents (e.g., vinflunine or a taxane). Triplet regimens should be avoided in patients with pre-existing neuropathy or low GFR. Thus, chemotherapy selection is tailored to performance status, renal function, and nodal stage; essentially, “chemotherapy-fit” patients with extensive lymph node disease receive cisplatin-based multi-agent regimens, whereas those with contraindications to cisplatin are directed toward less nephrotoxic alternatives. Overall, ECOG performance status, renal function (GFR), and the extent of lymph node involvement are the key factors guiding regimen selection.

To summarize the practical aspects of regimen selection, the EAU-ASCO guideline defines platinum eligibility and chemotherapy fitness as follows:

- Cisplatin-eligible: ECOG PS 0–1 and GFR > 50–60 mL/min.
- Carboplatin-eligible: ECOG PS 2 and GFR 30–60 mL/min.
- Platinum-unfit: ECOG PS > 2 or GFR < 30 mL/min.

In cisplatin-unfit but otherwise fit patients (ECOG PS ≤ 2, GFR ≥ 60 mL/min), vinflunine may be considered according to EAU-ASCO recommendations [8].

### 4.5. Future Directions in the Systemic Treatment of PSCC

Despite improvements in staging and multidisciplinary management, outcomes for advanced PSCC remain poor, and conventional platinum-based chemotherapy provides limited and short-lived benefit with significant toxicity [8]. Current systemic research therefore focuses on biomarker-driven immunotherapy, HER-family targeting, antibody–drug conjugates, and rational combinations, many of which are still only explored in small cohorts, basket trials, or early-phase studies [227,228,229].

Approximately half of PSCCs overexpress EGFR and other HER-family receptors, providing a biologically plausible target [227,228,229,230]. Small retrospective series and case reports with cetuximab, panitumumab, nimotuzumab, and tyrosine kinase inhibitors (erlotinib, gefitinib) have demonstrated objective responses in heavily pretreated or chemotherapy-ineligible patients, with response rates in the 30–50% range and clinically meaningful symptom control [228,229,231,232,233,234]. In addition, small neoadjuvant series of nimotuzumab combined with paclitaxel or platinum-based chemotherapy have reported major pathological responses, including complete responses, in locally advanced disease [56,231,232,233,234]. More systematic evaluation of HER inhibition comes from the phase II dacomitinib trial in N2–3/M1 PSCC, where first-line pan-HER blockade yielded an ORR of about 32% and median OS around 20 months, with acceptable tolerability [235,236]. These data, together with individual reports of durable responses to EGFR-directed antibodies after chemotherapy failure [231,232,237], support the continued exploration of HER-targeted strategies, either as monotherapy in frail patients or in combination regimens [233,234,238].

The inflammatory and virally driven biology of many PSCCs provides a strong rationale for immunotherapy. Tumor PD-L1 expression has been reported in roughly one-third to three-quarters of cases, and dense CD8+ T cell infiltrates and HPV positivity appear to correlate with improved outcome under PD-1/PD-L1 blockade [239,240,241,242,243,244,245,246]. In prospective phase II trials, first-line cemiplimab plus platinum chemotherapy (EPIC-A) and pembrolizumab with cisplatin/carboplatin–5-FU (HERCULES) achieved ORRs of approximately 40–50%, with median PFS around 5–6 months and median OS in the 12–16-month range [243,244,247,248]. Retrospective series and small real-world cohorts of chemo-immunotherapy have largely confirmed these response rates without novel safety signals, although follow-up remains short and patient numbers small [233,234,241,249].

Checkpoint inhibitor monotherapy has shown more modest but clinically relevant activity in pretreated or chemotherapy-ineligible patients. Single-agent PD-1/PD-L1 blockade with atezolizumab (PERICLES), retifanlimab (ORPHEUS), cemiplimab (EPIC-B and small retrospective series), avelumab (ALPACA), pembrolizumab, or nivolumab ± ipilimumab in basket trials has yielded ORRs in the 15–25% range and median PFS of approximately 3–5 months, with durable responses in a subset of HPV-positive or immunologically “hot” tumors [241,242,243,244,245,246,250,251,252,253]. Overall survival in these studies typically remains below one year, underscoring the need to better select patients and optimize combination strategies [243,244,245,246,249,251,252,253].

Roughly 50% of PSCCs are HPV-positive (predominantly HPV-16), and viral oncoproteins E6/E7 represent attractive tumor-specific antigens [242,243,244]. Case reports of dramatic and durable responses to PD-1 blockade in HPV16-positive, chemotherapy-intolerant patients, as well as exploratory analyses linking HPV status, p16 expression, and T cell infiltration with longer PFS under checkpoint inhibitors, support the development of HPV-specific vaccines and adoptive T cell therapies (TCR-modified T cells, tumor-infiltrating lymphocytes) in HPV-driven disease [243,244,245,246]. Early-phase studies of such HPV-targeted approaches in anogenital malignancies, including PSCC, are ongoing and may help refine biomarker-guided patient selection [237,242,246,254].

High expression of Nectin-4 and Trop-2 has been documented in sizeable subsets of primary and metastatic PSCC, with intermediate/high membranous Nectin-4 in roughly one-quarter of tumors and strong Trop-2 staining in the vast majority [250,255,256]. These data provide the biological basis for testing antibody–drug conjugates, particularly enfortumab vedotin (anti-Nectin-4) and sacituzumab govitecan (anti-Trop-2), which have already transformed the management of urothelial and other squamous cancers [249,250,255,256]. Dedicated phase II trials of enfortumab vedotin in metastatic PSCC and basket studies including penile cancer cohorts are underway and will clarify whether ADCs can offer chemotherapy-independent disease control in this population [249,250,255,256]. Additional targeted strategies, including combinations of VEGF/tyrosine kinase inhibition and PD-1 blockade, have shown signals of activity in small penile subsets within rare cancer basket trials, but responses have so far been transient [246,252,253].

Emerging data suggest that combining HER-targeted agents with ICIs and chemotherapy may further improve response rates. A single-center phase II trial of toripalimab (anti-PD-1), nimotuzumab (anti-EGFR), and taxane-based chemotherapy in locally advanced PSCC (TNT regimen) reported an ORR above 80% and encouraging 2-year PFS and OS, supporting the concept of multi-agent biochemotherapy in the neoadjuvant setting [238]. Similar multi-modality approaches integrating radiation, EGFR/HER blockade, checkpoint inhibition, and cytotoxic therapy are now being explored in early-phase protocols [229,233,234,238,249].

Notably, PSCC is rare, and progress depends on innovative trial designs and collaboration. Experts emphasize the need for global, multicenter networks and referral centers to enhance recruitment. Decentralized enrolment and even large retrospective cohorts have been proposed to make studies feasible. Likewise, concerted efforts to develop and share PSCC preclinical models (cell lines, organoids, or patient-derived iPSC models) are underway to accelerate drug testing. Taken together, these approaches—integrating biomarkers (EGFR, PD-L1, HPV status), novel agents, and coordinated research infrastructure—represent the future directions in the systemic treatment of PSCC [228,240,249,256]. An overview of completed and ongoing trials of biological and immune-based systemic therapies in PSCC is provided in Table 4.

## 5. Limitations

Despite the advances achieved through our research, which was based on a comprehensive summary of all available systemic chemotherapy regimens for PSCC identified across the searched databases along with their mechanistic contexts, this study has several limitations. Notably, the evidence base in PSCC remains weak. No randomized trials exist to definitively compare regimens; as a result, all compared guideline recommendations rely largely on retrospective series rather than prospective studies [8,9,10].

Most published reports across the decades are small, single-center, retrospective case series, often combining node-positive, locally advanced, and metastatic cases, which introduces heterogeneity in chemotherapy dosing, scheduling, and timing relative to surgery, making treatment effects vulnerable to bias, including a tendency to report only small positive experiences.

Although well-defined assessment criteria such as Response Evaluation Criteria in Solid Tumours (RECIST) are available [257], many studies do not apply them consistently, resulting in variable endpoint definitions and limiting the objective interpretation of response rates, survival outcomes, and long-term results. Toxicity data are similarly fragmentary and published rates vary widely [30,31,32,37,44,51,52,54].

The prognostic biomarkers identified to date, such as HPV status, routine laboratory parameters, and ECOG performance status, are derived from underpowered analyses. Some studies were unable to be evaluated due to the mixed results of various regimens [204,205,208,220,222,223,258,259,260,261] or even missing information about the regimens used [224,262].

The lack of high-level evidence has been emphasized in recent reviews: NCCN/EAU recommendations for neoadjuvant or adjuvant therapy were explicitly noted to rest on “low-level evidence” from limited cohorts. This deficiency extends to the salvage and second-line settings, where no standard systemic regimen is defined. In practice, most later-line treatments represent extrapolations from neoadjuvant regimens or from data in other squamous cell carcinomas [8,9,10].

In summary, the available literature is dominated by heterogeneous retrospective designs, small sample sizes, inconsistent reporting, and publication bias toward positive results, underscoring a critical need for robust, prospective, multicenter clinical trials to establish evidence-based systemic treatment strategies for PSCC. Therefore, any conclusions regarding chemotherapy efficacy or safety must be viewed with caution and validated in prospective trials.

## 6. Conclusions

Systemic chemotherapy using the TIP remains the reference neoadjuvant regimen for cisplatin-fit patients. For patients with reduced cisplatin fitness, carboplatin-based doublets (e.g., paclitaxel–carboplatin) or PF may be used. TPF is an alternative triplet option in cisplatin-eligible patients, but with higher toxicity.

Evidence for routine adjuvant chemotherapy is limited, and no definitive adjuvant protocol has been established. Advancing outcomes will require well-designed randomized trials in specialized centers and robust translational research to enable a true bench-to-bedside approach that links molecular insights to chemotherapy response and clinical decision-making. Identifying predictive biomarkers and evaluating novel treatments, including emerging biological therapies, will be essential in advancing personalized strategies, deepening our understanding of the response to combined systemic therapy and improving survival in this rare malignancy.

## Figures and Tables

**Table 1:** Evidence summary of chemotherapy regimens in PSCC—TIP/TPF/PF/TP (2000–2025).

Regimen	Is in 2023 EAU-ASCO Collaborative Guidelines [8] Preferred Approach?	Objective Response Rate (ORR), (%)	Partial Response Rate (PRR), (%)	Pathological Clinical Complete Response pCR/cCR (%)	Median Time to Progression (TTP), (Months)	Median Progression-Free Survival (PFS), (Months)	Median Overall Survival (OS), (Months)	Patient Enrollment Period (Years); Patients Evaluable for Response (*n*)	References
TIP (Paclitaxel/Ifosfamide/Cisplatin)	Yes, (neoadjuvant chemotherapy (NAC), adjuvant chemotherapy (AC), palliative)	50	40	pCR: 10	8.1	8.1	17.1	2000–2008 (*n* = 30)	Pagliaro et al. [35]
		60	60 (after 4 cycles)	0 (after 4 cycles)	n/a	n/a	6	2014–2016 (*n* = 17)	Sitompul et al. [36]
		low-stable (l-s): 62.5 high-decline (h-d): 37.5	(l-s): 62.5 (h-d): 37.5	pCR: (l-s): 51.9 (h-d): 21.2	n/a	(l-s): unreached (h-d): 10.3	(l-s): unreached (h-d): 23.2	2014–2022 (*n* = 80)	Ma et al. [43]
		100	100	0	n/a	n/a	n/a	2013–2017 (*n* = 1)	Dhasthakeer et al. [44]
		65	n/a	n/a	n/a	progressive disease: 7 stable disease: 31.5 complete/partial response: 25.5	progressive disease: 10.9 stable disease: 41.2 complete/partial response: 79.9	1993–2011 (*n* = 54)	Dickstein et al. [48]
		not applicable (n/a)	n/a	n/a	n/a	IIIb: 28 and 50 IV: 6	n/a	2008–2012 (*n* = 3)	O’Reilly et al. [49]
		80	0	cCR: 60	n/a	n/a	30	1985–2000 (*n* = 5)	Bermejo et al. [31]
TPF (Docetaxel/Cisplatin/5-Fluorouracil)	Yes, (NAC, AC, palliative)	38.5	38.5	0	n/a	3	7	2009–2013 (*n* = 39)	Zhang et al. [41]
		Locally advanced: 36.8 Distant metastases: 42.9 Per-protocol: 38.5	30.77	cCR: 7.69	n/a	Per-protocol: 6.9 1-year: Locally advanced: 48.8% Metastatic: 12.5%	Per-protocol: 7.7	2009–2010 (*n* = 26)	Nicholson et al. CRUK/09/001 [40]
		60	44	cCR: 8 pCR: 4	n/a	7 1-year: 31% 2-year: 12%	10.1 1-year: 46.2% 2-year: 26.9% disease-specific survival: 10.3	2008–2012 (*n* = 25)	Djajadini grat et al. [50]
T-PF (Docetaxel/Cisplatin/Fluorouracil): 85.7%; T-PF (Paclitaxel/Cisplatin/Fluorouracil): 14.3%	Yes, (Docetaxel–PF) (NAC, AC, palliative); No, (Paclitaxel–PF)	n/a	n/a	n/a	n/a	n/a	22.7, 1-year: 85%; disease-specific survival: 22.7, 1-year: 55.3%	2004–2012 (*n* = 21)	Necchi et al. [51]
T-PF (Docetaxel/Cisplatin/Fluorouracil): 76.47%; T-PF (Paclitaxel/Cisplatin/Fluorouracil): 23.53%		NAC: 58	NAC: 33.33 Metastatic: 17	cCR: NAC: 25 pCR: NAC: 17	n/a	NAC: 5 AC: 7 Metastatic: 2 In the whole series: 6	NAC: 5 AC: 9.5 Metastatic: 5 In the whole series: 6.5	2004–2010 (*n* = 34)	Salvioni et al. [38]
NAC/AC/Total [%] T-PF (Docetaxel/Cisplatin/Fluorouracil): 82/84/83; T-PF (Paclitaxel/Cisplatin/Fluorouracil): 18/16/17		NAC: 42.9	NAC: 39.3	pCR: NAC: 14	AC: 42 NAC: 17	38.3% remain disease-free after a median follow-up of 22 months The 2-year disease-free survivals: AC 36.8%; NAC 7.1% (On Kaplan–Meier Curves)	Unreached (On Kaplan–Meier Curves)	2004–2012 (NAC: *n* = 28) (AC: *n* = 19)	Nicolai et al. [39]
T-PF (Docetaxel/Cisplatin/Fluorouracil): 16.67%; T-PF (Paclitaxel/Cisplatin/Fluorouracil): 83.33%		Paclitaxel–PF: 80 Docetaxel–PF: 100	Paclitaxel–PF: 20	Paclitaxel–PF: pCR: 40; cCR: 20 Docetaxel–PF: pCR: 100	Paclitaxel–PF: 12 Docetaxel–PF: 0	n/a	Paclitaxel–PF: *n* = 3 NED (no evidence of disease), *n* = 3 median: 6 Docetaxel–PF: unreached, NED	2004–2006 (*n* = 6)	Pizzocaro et al. [37]
PF (Cisplatin/5-fluorouracil)	Yes, (NAC, palliative)	n/a	n/a	n/a	n/a	>20	n/a	2008–2012 (*n* = 1)	O’Reilly et al. [49]
		100	n/a	n/a	n/a	n/a	n/a	1972–2005 (*n* = 1)	Leijte et al. [52]
		57	42	cCR: 15	6	6	disease-specific survival: 22% (>24 mo)	2001–2004 (*n* = 27)	Chacko et al. [53]
		n/a	n/a	n/a	n/a	n/a	14 vs. 6 with surgery alone; disease-specific survival: 10	2010–2018 (*n* = 16)	Koifman et al. [54]
		32	32	0	n/a	~4.6	8	2000–2011 (*n* = 25)	Di Lorenzo et al. [55]
		NAC: 85.71	NAC: 85.71	NAC: 0	n/a	NAC: 7 AC: 6	n/a	2013–2017 (AC: *n* = 45) (NAC: *n* = 23)	Dhasthakeer et al. [44]
TP1 (Paclitaxel/Carboplatin)	Yes, (palliative)	NAC: 100	NAC: 33.33	NAC: cCR: 66.67	n/a	NAC: 10 AC: 10	n/a		
		100	100	0	n/a	n/a	n/a	2003 (*n* = 1)	Joerger et al. [30]
		50	50	0	n/a	n/a	67	1985–2000 (*n* = 2)	Bermejo et al. [31]
TP2 (Paclitaxel/Cisplatin)	No	n/a	n/a	n/a	n/a	n/a	n/a	2013–2017 (AC: *n* = 2)	Dhasthakeer et al. [44]
		0	0	0	6	n/a	n/a	2013 (published) (*n* = 1)	Pandey et al. [56]
TP1 Paclitaxel/Carboplatin: 21.05%; TP2 Paclitaxel/Cisplatin: 88.95%	Yes, Paclitaxel/Carboplatin (palliative); No, Paclitaxel/Cisplatin	n/a	n/a	n/a	n/a	median disease-free survival: overall: 16.2 treatment completed: 23.13 not completing treatment: 2.16	not reached	2008–2009 (*n* = 19: TP1: *n* = 4, TP2: *n* = 15)	Noronha et al. [32]
TP1 Paclitaxel/Carboplatin: 76.9%; TP2 Paclitaxel/Cisplatin: 23.1%	Yes, Paclitaxel/Carboplatin (palliative); No, Paclitaxel/Cisplatin	30.8	TP1: 30 TP2: 33.33	0	n/a	~3.1	~6.7	2008–2011 (*n* = 13, TP1 *n* = 10, TP2 *n* = 3 and refused chemotherapy *n* = 5)	Patil et al. [33]

**Table 2:** Mechanistic drug classes.

Class of Agent	Type	Subtype	Representative Drugs	Summary of Mechanism of Action	Notes/Key Toxicities	Report in PSCC
Alkylating Agents	Metal salts	-	Cisplatin	Forms DNA crosslinks that disrupt replication and transcription	Cisplatin intolerance in PSCC [69]	[20,26,27,28,29,31,32,33,35,36,37,38,39,40,41,43,44,48,49,50,51,52,53,54,55,56,94,103,104,107,108,118,120,129,182,191,198,199,200,201,202,203,204,205]
		-	Carboplatin		Better tolerance than cisplatin, reduced nephro- and neuro- toxicity [70,75]	[30,31,32,33,44,119,205]
		-	Oxaliplatin		One case report with no results noted	[56]
	Mustard gas derivatives	-	Ifosfamide	Alkylates DNA to form crosslinks that inhibit replication and transcription	Requires Mesna coadministration	[31,35,36,43,44,48,49,204]
Antimetabolites	Pyrimidine antagonists	Uracil analog	5-Fluorouracil (5-FU)	Inhibits the activity of TYMS, incorporation into nucleic acids, DNA damage	In PSCC only in combined regimens	[25,28,29,37,38,39,40,41,44,49,50,51,52,53,54,55,108,118,120,129,204,205]
		Cytosine analog	Gemcitabine	Incorporates into DNA causing chain termination and apoptosis	Upregulation of MHC I and PD-L1 and selective depletion of myeloid-derived suppressor cells [100]	[56,94,103,104,200,201,205]
		Pro-drug of 5-FU	Capecitabine	Converted to 5-FU within tumor tissue	Report from American Cancer Society [206]; reduced systemic exposure [197]	[56,204,206]
	Folic acid antagonists	-	Methotrexate	Inhibits dihydrofolate reductase, blocking tetrahydrofolate synthesis and DNA replication and repair	Leucovorin Rescue is strongly recommended [113]	[16,18,19,25,26,27,31,52,107,108,118,119,120,129,182,198,199,200,203,204,205,207]
Antitumor Antibiotics	Anthracyclines	-	Doxorubicin/Adriamycin	Intercalates into DNA and inhibits topoisomerase II, causing strand breaks and blocking replication	Risk of cumulative cardiotoxicity	[120,129]
		-	Epirubicin		Less cardiotoxicity compared with doxorubicin	[120,130,204]
		Doxorubicin analog	Pirarubicin		Less cardiotoxicity compared with doxo- and epirubicin; rarely used, reports only from Japan	[208,209]
	Chromomycins	-	Actinomycin D	Intercalates into DNA, blocking RNA polymerase and inhibiting transcription	Rarely used	[24]
	Miscellaneous	-	Bleomycin	Generates free radicals that cause DNA strand breaks, inhibiting replication and transcription	Strongly not recommended due to pulmonary toxicity	[15,19,21,23,24,25,26,27,31,52,107,108,118,120,129,182,198,199,200,203,204,205]
		Bleomycin analog	Peplomycin		Rarely used, reports only from Japan; Pulmonary toxicity comparable to bleomycin	[119,209,210,211]
		-	Mitomycin C	Alkylates and crosslinks DNA, blocking replication and transcription	Hypoxia-directed activity	[108,118,212]
Plant Alkaloids	Vinca alkaloids	-	Vincristine	Binds tubulin, inhibiting microtubule formation and blocking mitosis	Risk of vincristine-induced peripheral neuropathy (VIPN)	[19,23,25,52,213]
		-	Vinblastine		Significant myelosuppression	[24,119,120,205]
		-	Vinflunine		Single VinCaP Phase II trial in PSCC	[42]
		-	Vinorelbine		Lower neurotoxicity than other vinca alkaloids [162]	[56,200]
	Taxanes	-	Paclitaxel	Stabilizes microtubules, preventing their depolymerization and blocking mitosis	Risk of peripheral neuropathy, myelosuppression, and hypersensitivity reactions	[30,31,32,33,34,35,36,37,38,39,43,44,48,49,51,56,204,205]
		-	Docetaxel		Reduced risk of hypersensitivity reactions and neurotoxicity	[37,38,39,40,41,50,51,200,204]
		-	Cabazitaxel		Active in docetaxel-resistant tumors	[214]
	Podophyllotoxins	-	Etoposide	Topoisomerase II inhibitor, causing DNA strand breaks and blocking replication and transcription	One case report with no response noted (combined with cisplatin and methotrexate)	[130]
	Camptothecan analogs	-	Irinotecan	Topoisomerase I inhibitor, causing DNA strand breaks and blocking replication and transcription	Single CPT 11 Phase II trial in PSCC	[191]

**Table 3:** Indications and preferred regimens for systemic chemotherapy in PSCC.

Category	Aspect	EAU-ASCO 2023 [8]	ESMO-EURACAN 2024 [9]	NCCN 2.2025 [10]
Neoadjuvant Chemotherapy	Indications	Chemotherapy-fit patients with pelvic lymph node involvement, extensive inguinal involvement (cN3), bulky/bilateral mobile inguinal nodes (cN2).	Locally advanced/high-risk PSCC; bulky, fixed, ulcerated, or cN3 nodes; locally advanced T4 inoperable tumors.	TX, N2–3, M0 PSCC; unilateral mobile nodes ≥4 cm, fixed nodes, bilateral nodes with positive fine-needle aspiration (FNA)/biopsy; enlarged pelvic nodes; unresectable T4 tumors.
	Preferred Regimens	Cisplatin- and taxane-based combinations (TIP, TPF), doublet or triplet preferred.	TIP or TPF triplet regimens preferred.	TIP only; PF not recommended.
Adjuvant Chemotherapy	Indications	Pathological pelvic lymph node involvement (pN3) post-LND without prior NAC; weak recommendation.	High-risk, node-positive pN2/pN3 post-LND (≥2 positive nodes, extranodal extension, metastasis >30 mm, or pN3 post-LND).	Post-LND inguinal lymph node-positive disease with high-risk features (pelvic lymph node metastases, extranodal extension, bilateral inguinal nodes involvement, lymph node tumors ≥4 cm); if ≥2 positive nodes or extranodal extension.
	Preferred Regimens	Cisplatin- and taxane-based (doublet/triplet): TIP, TPF. (consistent with those used in NAC)	Triplet regimens: TIP, TPF (mirroring neoadjuvant settings) combined with radiotherapy (RT) for pN3.	TIP preferred (extrapolation from neoadjuvant data); PF (cisplatin + 5-FU) as alternative.
	Evidence Level	Limited, conflicting; weak recommendation.	Limited to retrospective cohorts; combined chemotherapy (ChT) + RT improves cancer specific survival (CSS) (29% vs. 16% for ChT alone in pN3).	Insufficient data; extrapolated from neoadjuvant TIP data.
Palliative Chemotherapy	Indications	Advanced/metastatic or inoperable disease (M1)	Metastatic, inoperable, or recurrent PSCC; based on comorbidities, ECOG status.	Metastatic or recurrent PSCC with distant metastases.
	First-Line Regimens	Platinum-based chemo triplet (TPF, TIP) or doublet (cisplatin+5-FU (PF), paclitaxel/carboplatin); single-agent for platinum-unfit patients (vinflunine or taxanes); bleomycin not recommended.	Platinum-based combinations (TIP, TPF, PF, carboplatin + paclitaxel);	TIP preferred; alternatives: PF or 5-FU + pembrolizumab + cisplatin/carboplatin; bleomycin not recommended.
	Second-Line Options	Poor outcomes (OS ≤6 months); No effective regimens.	Limited; suggests systemic ChT (no regimen), RT, or clinical trials.	Limited; prefers clinical trials, paclitaxel, or chemoradiotherapy (CRT) (cisplatin alone; PF; mitomycin C + 5-FU; capecitabine) or tumor-agnostic therapies.

**Table 4:** Clinical trials of biological and immune-based systemic therapies in penile squamous cell carcinoma (only studies reporting the number of enrolled PSCC patients).

Title	Treatment	Area of Investigation	Studied Population	Status	Registration Number	No. of Enrolled PSCC Patients
Phase II Basket Trial Evaluating the Efficacy of a Combination of Pembrolizumab and Vorinostat in Patients with Recurrent and/or Metastatic Squamous Cell Carcinoma	Pembrolizumab and Vorinostat	PD-1	Mucosal cancers	Active, not recruiting	NCT04357873	*n* = 11
A Multicenter, Open-Label, Single-Arm, Phase II Clinical Trial to Evaluate the Efficacy and Safety of INCMGA00012 in Advanced Penile Squamous Cell Carcinoma (ORPHEUS)	Retifanlimab	PD-1	Penile Cancer	Completed	NCT04231981	*n* = 18
A Phase II Trial of Pembrolizumab Combined With Cisplatin-based Chemotherapy as First-line Systemic Therapy in Advanced Penile Cancer (HERCULES)	Pembrolizumab	PD-1	Penile Cancer	Completed	NCT04224740	*n* = 33
A phase II trial of cemiplimab alone or in combination with standard of care chemotherapy in locally advanced or metastatic penile carcinoma (EPIC Trial)	Cemiplimab with platinum-based chemotherapy or in monotherapy	PD-1	Penile Cancer	Completed	NCT95561634	*n* = 47
Phase II Study for the Evaluation of Efficacy of Pembrolizumab (MK-3475) in Patients With Rare Tumors	Pembrolizumab	PD-1	Rare tumors	Active, not recruiting	NCT02721732	*n* = 3
A Phase II Study of Nivolumab Combined With Ipilimumab for Patients With Advanced Rare Genitourinary Tumors	Nivolumab and Ipilimumab	PD-1, CTLA-4	Advanced Rare Genitourinary Tumors	Active, recruiting	NCT03333616	*n* = 6
A Single-Center and Single-Arm Study of TIP (Paclitaxel + Ifosfamide + Cisplatin) Combined with Nimotuzumab and Triprilimab as Neoadjuvant Treatment in Locally Advanced Penile Cancer	Nimotuzumab and Triprilimab	PD-1,EGFR	Penile cancer	Completed	NCT04475016	*n* = 29
A Phase 1 Study of Cabozantinib Plus Nivolumab (CaboNivo) Alone or in Combination With Ipilimumab (CaboNivoIpi) in Patients With Advanced/Metastatic Urothelial Carcinoma and Other Genitourinary Tumors	Cabozantinib and nivolumab with or without ipilimumab	PD-1,VEGFR, CTLA-4	Metastatic Genitourinary Tumors	Active, recruiting	NCT02496208	*n* = 9
PERICLES (PEnile Cancer Radio- and Immunotherapy CLinical Exploration Study)-a Phase 2 Study of Atezolizumab With or Without Radiotherapy in Penile Cancer	Atezolizumab	PD-L1	Penile Cancer	Completed	NCT03686332	*n* = 32
Phase II Study of the Pan-HER Inhibitor Dacomitinib (PF-00299804) for Patients With Locally Advanced or Metastatic Squamous Cell Carcinoma of the Penis	Dacomitinib	Pan-HER	Penile Cancer	Completed	NCT01728233	*n* = 32

## Data Availability

No new data were created or analyzed in this study. Data sharing is not applicable to this article.

## Supplementary Materials

The following supporting information can be downloaded at https://www.mdpi.com/article/10.3390/cancers18010046/s1, Table S1: Evidence summary of chemotherapy regimens in PSCC—TIP/TPF/PF/TP (2000–2025)—a broader context; Table S2: Evidence summary of chemotherapy regimens in PSCC—bleomycin-containing, MTX monotherapy, and other historical regimens (1965–2000); Table S3: Evidence summary of chemotherapy regimens in PSCC—emerging, second-line, and other regimens; Text S1: Mechanistic drug classes—a broader context. References [8,15,16,18,19,20,21,23,25,26,27,28,29,30,31,32,33,34,35,36,37,38,39,40,41,42,43,44,46,47,48,49,50,51,52,53,54,55,56,57,58,59,60,61,62,63,64,65,66,67,68,69,70,71,72,73,74,75,76,77,78,79,80,81,82,83,84,85,86,87,88,89,90,91,92,93,94,95,96,97,98,99,100,101,102,103,104,105,106,107,108,109,110,111,112,113,115,116,117,118,119,120,121,122,123,124,125,126,127,128,129,130,131,132,133,134,135,136,137,138,139,140,141,142,143,144,145,146,147,148,149,150,151,152,153,154,155,156,157,158,159,160,161,162,163,164,165,166,167,168,169,170,171,172,173,174,175,176,177,178,179,180,181,182,183,185,186,187,188,189,190,191,192,193,194,195,196,197,198,199,200,201,202,203,204,207,209,210,211,212,213,214] are cited in the Supplementary Materials.

## Abbreviations

The following abbreviations are used in this manuscript:

5-FU	5-fluorouracil
AC	adjuvant chemotherapy
ADCs	antibody–drug conjugates
APC	adenomatous polyposis coli
ASCO	American Society of Clinical Oncology
BMP	cisplatin + methotrexate + bleomycin
CBDCA	cyclobutane-1,1-dicarboxylate
ChT	chemotherapy
CR	complete response
cCR	clinical complete response
CRediT	contributor roles taxonomy
CRT	chemoradiotherapy
CSS	cancer specific survival
cN/pN	clinical/pathological nodal status
cT/pT	clinical/pathological tumor (primary) status
CYP3A4	cytochrome P450 family 3 subfamily A member 4
DHFR	dihydrofolate reductase
DNA	deoxyribonucleic acid
DSNB	dynamic sentinel node biopsy
EAU	European Association of Urology
ECOG PS	Eastern Cooperative Oncology Group Performance Status
EGFR	epidermal growth factor receptor
ENE	extranodal extension
ESMO	European Society for Medical Oncology
EURACAN	European Reference Network for Rare Adult Solid Cancers
FDG-PET/CT	fluorodeoxyglucose positron emission tomography/computed tomography
GC (GP)	gemcitabine + cisplatin (gemcitabine + platinum)
G-CSF	granulocyte colony-stimulating factor
GFR	glomerular filtration rate
HER	human epidermal growth factor receptor
HR	hazard ratio
HPV	human papillomavirus
ICI	immune checkpoint inhibitor
IHC	immunohistochemistry
ILND/PLND/mILND	inguinal/pelvic lymph node dissection/modified ILND
i.m.	intramuscular
iPSCs	induced pluripotent stem cells
i.v.	intravenous
MRI	magnetic resonance imaging
MTX	methotrexate
NAC	neoadjuvant chemotherapy
NCCN	National Comprehensive Cancer Network
NCI	National Cancer Institute
ORR	objective response rate
OS	overall survival
pCR	pathological complete response
PD-1/PD-L1	programmed cell death protein 1/ligand 1
PF	cisplatin + 5-fluorouracil
PFS	progression-free survival
PR	partial response
PSCC	penile squamous cell carcinoma
PTEN	phosphatase and tensin homolog
QoL	quality of life
RECIST	Response Evaluation Criteria in Solid Tumors
RNA	ribonucleic acid
RT	radiotherapy
SCC	squamous cell carcinoma
SCC-Ag	squamous cell carcinoma antigen
SD	stable disease
SMAD4	mothers against decapentaplegic homolog 4
SWOG	Southwest Oncology Group
TCR	T cell receptor
TEAE/TRAE	treatment-emergent/treatment-related adverse event
TIL	tumor-infiltrating lymphocytes
TIP	paclitaxel + ifosfamide + cisplatin
TNM	tumor–node–metastasis (AJCC/UICC staging)
TP	taxane (docetaxel or paclitaxel) + platinum
TPF	taxane + cisplatin + 5-FU
Trop-2	trophoblast cell surface antigen-2
TTP	time to progression
TYMS	thymidylate synthase
ULN	upper limit of normal
VFL	vinflunine
VBM	vincristine + bleomycin + methotrexate
