# Supplementary material for: Systemic Chemotherapy in Penile Squamous Cell Carcinoma: Mechanisms, Clinical Applications, and Evidence-Based Regimens
Source: Cancers (Basel). 2025 Dec 23;18(1):46. doi: 10.3390/cancers18010046 (PMC12785095; doi:10.3390/cancers18010046)
Supplement: Supplementary file 1 [file cancers-18-00046-s001.zip › Table S1.pdf]

Table S1: Evidence summary of chemotherapy regimens in PSCC—TIP/TPF/PF/TP (2000-2025)—a broader context									
**Regimen**	**The treatment regimen**	**Completion of Planned Cycles**	**Indication, staging or TNM classification system**	**Toxicity. (If Available: Common Terminology Criteria for Adverse Events (CTCAE) [46]; (G= Grade)**	**If simultaneous with other non-surgical therapy?**	**Study Design, (patients evaluable for response)**	**Level of Evidence the Oxford Centre for Evidence-Based Medicine. (OCEBM) criteria [47]**	**Patient Enrollment Period (years)**	**References**
TIP (Paclitaxel/Ifosfamide/Cisplatin)	four cycles of 21- to 28-days duration each: 175 mg/m2 paclitaxel intravenously (IV) over 3 hours day 1; 1,200 mg/m2 ifosfamide IV over 2 hours days 1, 2, and 3; and 25 mg/m2 cisplatin IV over 2 hours days 1, 2, and 3. The cycle was repeated on day 22 if the patient's absolute neutrophil count was at least 1,400/µL and platelet count was at least 100,000/µL.	n=23 (76.7%) completed the planned 4 courses n=7 discontinued chemotherapy after 1 to 3 courses; the reasons were: n=3: rapid tumour progression, n=1: hypersensitivity to paclitaxel, n=1: cardiac event, n=2: patient's decision.	NAC; PSCC; T [any] stage; clinical stage N2 or N3; M0	G3: Central venous catheter-related thrombosis, Deep vein thrombosis, Hyperglycemia, Infection, Myocardial ischemia, Anemia, Febrile neutropenia, Motor neuropathy, Thrombocytopenia G4: Allergic reaction, Neutropenia	No	Prospective, single-institution, nonrandomized, Phase II (n=30)	II	2000-2008	Pagliaro et al. [35]
	four cycles of: paclitaxel 175 mg/m2 on day 1, ifosfamide 1200 mg/m2 on day 1 to 3, and cisplatin 25 mg/m2 on day 1 to 3 with duration of 21-28 days.	10 patients completed the full 4 cycles of the regimen. n=4: died before completion, n=2: refused to continue, n=1: still on the second cycle.	NAC, AC; PSCC; T1b,2,3,4, x stage; N1,2,3 stage; M0	Neutropenia, Thrombocytopenia, Anaemia, Mucositis, Nausea, Diarrhoea, Alopecia	No	Retrospective, single-institution, (n=17)	IV	2014-2016	Sitompul et al. [36]
	albumin-bound paclitaxel 260 mg/m2, Day 1; cisplatin 25 mg/m2, Days 1–3, ifosfamide1200 mg/m2, Days 1–3; intravenously every 3 weeks, with no more than 5 cycles, or until dose-limiting toxicity or disease progression or patient withdrawal.	median cycles: 4 (3-4 in each group).	NAC, AC, Palliative. Two trajectories of SCC-A, TNM Staging: III/IV: low-stable (l-s) (40%; n = 32); high-decline (h-d) (60%; n = 48)	n/a	n=60 (75%) patients received combined use of targeted and immune therapies: TIP+Anti- PD1 + anti- EGFR; nimotuzumab 400 mg and toripalimab 240 mg were injected intravenously on Day 1.	Retrospective, single-institution, (n=80)	III	2014–2022	Ma et al. [43]
	Paclitaxel (260 mg/m² on Day 1), Ifosfamide (1.2 gm/m² from Day 1 to Day 3) and Cisplatin (25 mg/m² from Day 1 to	Yes	NAC; TNM Staging: IIIa	p>0,05: Diarrhoea, Abdominal Pain, Fever	No	Prospective or Retrospective	III	2013-2017	Dhasthakeer et al. [44]

	Day 3);<br>six chemotherapy cycles, spaced every three weeks					single-institution<br>(n=1)			
	n/a	median cycles: 4 (range 1-10),<br>median with a response: 4<br>median with progression: 5	NAC;<br>T[any] N1-3 M0	no patient succumbed due to chemotherapy<br>or was deemed unfit for surgery related<br>to chemotherapy toxicity.	Results were measured for cohort of 60 responsive patients. Seven patients<br>received other initial chemotherapy combinations than TIP.<br>(Five of seven: carboplatin/paclitaxel or 5 fluorouracil/cisplatin, or methotrexate/bleomycin/cisplatin)	Retrospective,<br>single-institution,<br>(n=54)	III	1993-2011	Dickstein et al.<br>[48]
	paclitaxel 175 mg/m2 on day 1;<br>ifosfamide 1,200 mg/m2 on days 1-3;<br>and cisplatin 20 mg/m2on days 1-3.<br>All pts received 4 X 3 weekly cycles	Yes	AC;<br>pT3N2M0 (stage IIIB)<br>pT1bN3M0 (stage IV)	no significant toxicity	No	Retrospective,<br>single-institution,<br>(n=3)	III	2008-2012	O'Reilly et al.<br>[49]
	On day 1 175 mg/m2 paclitaxel i.v. for 5 days.<br>On days 1 to 3, 1.2 gm/m2 ifosfamide i.v. and 20 mg/m2 cisplatin i.v.;<br>21 days for a total of 4 or 5 cycles.	Yes	NAC;<br>T1,3,xN1,3M0	no toxic-related deaths	No	Retrospective (n=5)	IV	1985-2000	Bermejo et al.<br>[31]
TPF<br>(Docetaxel/Cisplatin/5-Fluorouracil)	docetaxel 75 mg/m2 (1hour i.v. infusion),<br>cisplatin 70 mg/m2 (1 to 3hour i.v. infusion) on day 1,<br>fluorouracil 500 mg/m2/d (continuous i.v. infusion)<br>for 5 days every 3 weeks.	median of 4 cycles (range 2 to 6)	Palliative;<br>T[any]N[any]M1	G3-4:<br>Anemia, Neutropenia, Thrombocytopenia, Febrile neutropenia,<br>Central vein catheter related thrombosis;<br>Nausea/vomiting, Diarrhea, Infection, Fever, Mucositis,<br>Deep vein thrombosis, Neuropathy sensor	No	Prospective,<br>single-institution,<br>openlabel,<br>Phase II<br>(n=39)	II-III	2009-2013	Zhang et al.<br>[41]
	Docetaxel 75 mg m2 day 1 i.v. over 1 h;<br>Cisplatin 60 mg m2 day 1 i.v. over 2 h;<br>5-fluorouracil 750 mg m2 per day as a continuous i.v. infusion<br>over days 1–5 (total dose 3750 mg m2).<br>Three cycles with a cycle time of 21 days.	overall mean relative dose intensity: 84%.<br>n=21 (72.4%) all 3 cycles ;<br>n=5 (17.2%) discontinued after 2 cycles;<br>n=3 (10.3%, all non-evaluable)<br>after a single cycle.	NAC/Palliative;<br>M1; T[any]N3M0;<br>T[any]N2M0;<br>T3N1M0;<br>T4N[any]M0	67.9% of patients experienced one or more grade 3 or<br>4 toxicity during treatment.<br>There were no toxic deaths<br>G3-4:<br>Neutropenia, Sepsis, Diarrhoea , Febrile neutropenia,<br>Leucopenia, Anaemia,	No	Multi-centre,<br>single-arm,<br>Phase II<br>(n=26)	II	2009-2010	Nicholson et al.<br>CRUK/09/001<br>[40]

		n=18 had a dose reduction or delay;<br>reasons:<br>n=5: toxicity;<br>n=8: clinical decision<br>n=1: patient choice;<br>n=4: administrative error.<br>4 dose reductions:<br>(docetaxel dose reduced from 75 to 60 mg m2)<br>resulted from haematological toxicity.		Asthenia, Nausea, Peripheral oedema, Syncope					
	75 mg/m2 docetaxel (day 1),<br>75 mg/m2 cisplatin (day 1),<br>and 750 mg/m2 5-fluorouracil (days 1-4)<br>every 3 weeks, 4 courses	n=12: (48%) all 4 cycles.<br>n=4: (15%) received modified cycles because of toxicity<br>(dose modification or replacement<br>of cisplatin by carboplatin);<br>n=10 (38%) did not complete all cycles<br>because of:<br>n=7: disease progression;<br>n=2: toxicity;<br>n=1: both.	NAC;<br>Tx,2 N2,3 M0;<br>T4N0M0	G2:<br>Anemia, Acute coronary syndrome, Atrial fibrillation, Chest pain (cardiac),<br>Diarrhea, Nausea, Oral mucositis, Edema limbs, Fatigue/malaise,<br>Infusion site extravasation, Infusion site reaction, Allergic reaction,<br>Hypocalcemia, Hypokalemia,<br>Hypomagnesemia, Dysgeusia,<br>Acute kidney injury;<br>G3:<br>Anemia, Febrile neutropenia,<br>Heart Failure, Nausea,<br>Hypokalemia, Hypomagnesemia, Syncope, Acute kidney injury.<br>G4:<br>Abdominal infection, Low Neutrophil count	No, only subsequent	Prospective, nonrandomized, single institution trial.<br>(n=25)	III	2008-2012	Djajadiningrat et al.<br>[50]
T-PF<br>(Docetaxel/Cisplatin/Fluorouracil): 85,7%;<br>T-PF<br>(Paclitaxel/Cisplatin/Fluorouracil): 14,3%	the 2 following schedules:<br>1. paclitaxel 120 mg/m2 on day 1,<br>cisplatin 100 mg/m2 on day 1,<br>and 5-FU 1000 mg/m2 on day 1,<br>96-hour i.v. infusion, every 3 weeks;<br>2. docetaxel 75 mg/m2 on day 1,<br>cisplatin 75 mg/m2 on day 1,<br>5-FU on 750 mg/m2 on day 1,<br>96-hour i.v. infusion, every 3 weeks.<br>A maximum of 4 cycles.	Cycles:<br>2: 23,8 %<br>3: 66,7 %<br>4: 9,5 %	AC;<br>cN+ before lymphadenectomy.	G3-4 neutropenia (n=4) and G3 thrombocytopenia (n=1).<br>No deaths from toxicity occurred.	No	Retrospective, single-institution,<br>(n=21)	IV	2004-2012	Necchi et al.<br>[51]
T-PF<br>(Docetaxel/Cisplatin/Fluorouracil): 76,47%;<br>T-PF	3-4 courses of paclitaxel 120 mg/m2<br>or docetaxel 75 mg/m2 d1 + cisplatin 75 mg/m2<br>+ 5-fluorouracil (5FU)<br>96 hrs continuous infusion 750 mg/m2	n/a	NAC cN2/3 M0;<br>AC pN2-3;<br>Metastatic: M1	G ≥ 3 renal and neurotoxicity	No	Prospective, single-center,<br>pilot trial.<br>(n=34)	III	2004-2010	Salvioni et al.<br>[38]

(Paclitaxel/Cisplatin/Fluorouracil): 23,53%									
NAC/AC/Total [%]<br>T-PF (Docetaxel/Cisplatin/Fluorouracil): 82/84/83;<br>T-PF (Paclitaxel/Cisplatin/Fluorouracil): 18/16/17	up to 4 cycles every 21 days with 120 mg/m2 paclitaxel I.V. over 1 hour on day 1;<br>100 mg/m2 cisplatin I.V. over 2 hours of infusion on day 1;<br>followed by 96 hours of continuous I.V. infusion of 1000 mg/m2/d of 5-fluorouracil.<br>Since January 2007, 75 mg/m2 of docetaxel replaced paclitaxel and PF was reduced to 75 and 750 mg/m2, respectively.	Median Courses:<br>NAC: 3 (3-3);<br>AC: 4 (2-4)	NAC cN2,3M0;<br>AC: pN2,3M0	One early death (< 30 days) due to cardiac treatment-related toxicity occurred in 1 patient in the NAC group.<br><br>NAC G≥3: anemia, neutropenia, thrombocytopenia, febrile neutropenia, alopecia, stomatitis (mucositis), diarrhea, neurotoxicity (peripheral sensory neuropathy), nephrotoxicity, cardiac toxicity, toxic death.<br><br>AC G≥3: anemia, neutropenia, thrombocytopenia, alopecia, stomatitis (mucositis), diarrhea.<br><br>Neutropenia was the most common toxicity in both groups.	No	Retrospective,<br>NAC: (n=28)<br>AC: (n=19)	III	2004-2012	Nicolai et al. [39]
T-PF (Docetaxel/Cisplatin/Fluorouracil): 16,67%;<br>T-PF (Paclitaxel/Cisplatin/Fluorouracil): 83,33%	120 mg/m2 paclitaxel on day 1;<br>followed by 50 mg/m2 cisplatin repeated on days 1 and 2;<br>and continuous i.v. infusion of 1000 mg/m2 5FU daily on days 2–5;<br>or in n=1: docetaxel (75 mg/m2), cisplatin, and 5FU	Paclitaxel-PF:<br>2 cycles in n=4 and 5 cycles in n=1;<br>Docetaxel-PF:<br>7 cycles in n=1	NAC,<br>G1T2N0, G3T3N3, G3T3N2, G3TxN3,G2T2N1, G2T1N2,	n=1: severe nausea and vomiting during TPF administration; hematologic toxicity did not exceed G2.	No	Retrospective, single-institution, case-series (n=6)	IV	2004-2006	Pizzocaro et al. [37]
PF (Cisplatin/5-fluorouracil)	4 cycles of cisplatin and 5-Fluorouracil	Yes	AC,<br>pT1N2M0	n/a	No	Retrospective, single-institution, (n=1)	IV	2008-2012	O'Reilly et al. [49]
	5-FU 1000 mg/m2 iv days 1–5;<br>Cisplatin 100 mg/m2 iv day 1,<br>repeated with a 3-wk interval until maximum of 5 cycles	n/a	NAC;<br>irresectable disease:<br>T1-4 N0,1,3, M0	n/a	No	Retrospective, single-institution, (n=1)	IV	1972-2005	Leijte et al. [52]
	cisplatin 70 mg/m2, given on day 1, and continuous infusion 5-Flurouracil (800 mg/m2 for 96 hours; days 1-4).<br>Carboplatin was substituted for cisplatin in 2 cases due to drop in GFR	A median of 5 cycles.	AC/Palliative;<br>59,3%: pN3<br>40,7%: M1	41%: any G3/4;<br>18,5%: G3/4 neutropenia;<br>7,4%: febrile illnesses requiring hospital admission and antibiotics;<br>7,4%: drop in GFR G2,	No	Retrospective, single-institution, (n=27)	III	2001-2004	Chacko et al. [53]

				probably related to Cisplatin (Carboplatin was substituted for cisplatin in these cases)<br>7,4%: G3 chemotherapy-induced nausea and vomiting and required hospitalization.					
	continuous infusion of 5-FU 800-1000mg/m2/day I.V. on days 1-4;<br>cisplatin 70-80mg/m2 IV on day 1<br>three courses, every 21 days	Yes	AC;<br>stage IV	n/a	No	Prospective, single-center,<br>(n=16)	III	2010-2018	Koifman et al. [54]
	cisplatin at a median dose of 75 mg/m2 (70-80) on day 1;<br>5-FU continuous infusion at the median dose of 900 (800-1000) mg/m2 for 4 days after cisplatin infusion in all patients every 21 days (one cycle).	Dose reductions in 28%.<br>Median 6 (4 – 6) cycles.	Palliative;<br>inoperable, stage IV	G1-4: Neutropenia, Anaemia, Thrombocytopenia;<br>G1-3: Oral mucositis, Nausea/vomiting, Peripheral neuropathy, Constipation, Alopecia, Diarrhoea;<br>G1-2: Hypercreatininaemia.<br>There was no death or interruption of treatment for toxicity.	No	Retrospective;<br>multicentre,<br>(n=25)	III	2000-2011	Di Lorenzo et al. [55]
	5-FU (1 gm/m² on Day 1);<br>cisplatin (75 mg/m² on Day 2),<br>six chemotherapy cycles,<br>spaced every three weeks	Yes	NAC/AC;<br>Stage: II,IIIa,IIIb,IV	G1-4: Anaemia,Thrombocytopenia,<br>Leukopenia, Neutropenia, Lymphocytopenia<br>The grade I-III haematological toxicity of anaemia, lymphocytopenia and thrombocytopenia was observed more in FP than TP1 and TP2 combinations	No	Prospective or Retrospective<br>AC (n=45)<br>NAC (n=23)	III	2013-2017	Dhasthakeer et al. [44]
TP1<br>(Paclitaxel/Carboplatin)	paclitaxel (175 mg/m² on Day 1);<br>carboplatin (450 mg/m² on Day 2),<br>six chemotherapy cycles,<br>spaced every three weeks	Yes	Neo/AC stage: II,IIIa,IIIb	Better tolerability than TIP<br>G1-4: Anaemia,Thrombocytopenia,<br>Leukopenia, Neutropenia, Lymphocytopenia	No	Prospective or Retrospective<br>AC (n=7)<br>NAC (n=5)			
	paclitaxel 75 mg/m2;<br>carboplatin AUC3<br>on days 1, 8, and 15<br>every 28 days.	3 cycles	NAC;<br>Recurrent, inguinal nodal metastasis.	Well tolerated.<br>G1 dysgeusia, G2 peripheral paresthesia, total alopecia.	No	Retrospective,<br>case report,<br>(n=1)	IV	2003	Joerger et al. [30]
	day 1 paclitaxel i.v. at a dose of 80 to 200 mg/m2;<br>carboplatin at a dose of AUC 6 (mg/minutes)/ml;<br>A total of 4 cycles.	Yes	NAC;<br>TxN1,2M0	no toxic-related deaths	No	Retrospective (n=2)	IV	1985-2000	Bermejo et al. [31]

TP2 (Paclitaxel/Cisplatin)	paclitaxel (175 mg/m² on Day 1); cisplatin (75 mg/m² on Day 1 and Day 2); six chemotherapy cycles, spaced every three weeks	Yes	AC; TNM Staging: IIIb	p>0,05: Vomiting, Constipation, Oral ulcer, Tumor Pain, Fever, Tumor ulceration, Mucositis, Penile skin infection/ ulceration	No	Prospective or Retrospective AC (n=2)	IV	2013-2017	Dhasthakeer et al. [44]
	n/a	3 cycles	AC; multiple groin node involvement and perinodal extension.	n/a	No	Retrospective, case report, (n=1)	IV	2013 (published)	Pandey et al. [56]
TP1 Paclitaxel/Carboplatin: 21,05%; TP2 Paclitaxel/Cisplatin: 88,95%	paclitaxel (175 mg/m2 over 3 hour); cisplatin (75 mg/m2 over 1 hour); In 21,05% carboplatin was substituted for cisplatin (due to serum creatinine clearance <50) 4 cycles were planned at an interval of 21 days in all patients.	63,2% completed planned 4 courses. median number of cycles: 4.	AC; population with high-risk features of local recurrence; any TNM, stage II-IV	1 toxic-related death (diarrhea and vomiting with febrile neutropenia); G1-3: vomiting; G2: diarrhea; G3: hearing loss; G1,2: neutropenia; G1-3: myalgia.	No	Retrospective, single-center, (n=19, TP1 n=4,TP2 n=15)	IV	2008-2009	Noronha et al. [32]
TP1 Paclitaxel/Carboplatin: 76,9%; TP2 Paclitaxel/Cisplatin: 23,1%	paclitaxel in a dose of 175 mg/m2 infusion over 3 hours; carboplatin at a dose of 5 times the AUC (area under curve) or cisplatin over 3 days at a dose of 25 mg/m2 per day in an infusion over 1-2 hours; administered once every 21 days.	The median number of cycles: 3 (1-6) TP1: no. of cycles: 1 cycle n=2 3 cycles n=3 4 cycles n= 1 6 cycles n=4 TP2 no. of cycles: 2 cycles n=1 3 cycles n= 1 4 cycles n=1	Palliative; metastatic disease in 38,9%, locally advanced disease in 61,1%. any TNM	nausea G1-3; vomiting G1-3; loose motion G2; sensory neurotoxicity G2; neutropenia G1,2; febrile neutropenia G3; myalgia G1-3. no toxicity related death. n=1 discontinued chemotherapy due to toxicity.	Yes, the response to chemotherapy, was consolidated by radiation in 3 patients.	Retrospective, single-center, (n=13, TP1 n=10, TP2 n=3) and n=5 refused chemotherapy (included in PFS and OS)	IV	2008-2011	Patil et al. [33]
