# Supplementary material for: Systemic Chemotherapy in Penile Squamous Cell Carcinoma: Mechanisms, Clinical Applications, and Evidence-Based Regimens
Source: Cancers (Basel). 2025 Dec 23;18(1):46. doi: 10.3390/cancers18010046 (PMC12785095; doi:10.3390/cancers18010046)
Supplement: Supplementary file 1 [file cancers-18-00046-s001.zip › Table S2.pdf]

Table S2: Evidence summary of chemotherapy regimens in PSCC—bleomycin-containing, MTX monotherapy and other historical regimens (1965–2000)																
**Regimen**	**The treatment regimen**	**Completion of Planned Cycles**	**Is in 2023 EAU-ASCO Collaborative Guidelines [8] preferred approach?**	**Indication, staging or TNM classification system**	**Objective Response Rate (ORR), (%)**	**Partial Response Rate (PRR), (%)**	**Pathological/Clinical complete response pCR/cCR (%)**	**Median time to progression (TTP), (months)**	**The median progression-free survival (PFS), (months)**	**the median overall survival (OS), (months)**	**Toxicity. (If Available: Common Terminology Criteria for Adverse Events (CTCAE) [46]; (G= Grade)**	**If simultaneous with other non-surgical therapy?**	**Study Design, (patients evaluable for response)**	**Level of Evidence the OCEBM criteria [47]**	**Patient Enrollment Period (years)**	**References**
PF (Cisplatin/5-fluorouracil)	intra-arterial chemotherapy (cisplatin 100 mg/m2 on day 1 + 5-fluorouracil 960 mg/m2 on days 2-6) in 3 cycles	3 courses	Yes, (NAC, palliative)	Adjuvant, second line; T2N3M0, stage IV	100	100	0	n/a	n/a	n/a	n/a	Yes, radiation: a total dose of 72 Gy to the inguinal lesions	retrospective, case report (n=1)	IV	1994-1999	Huang et al. [129]
	cisplatin intravenously at a dose of 100 mg/m2. 24 hours later a continuous intravenous	No. of cycles: 4,4,3,2,4		Jackson Stage 4: neoadjuvant n=1 Jackson Stage: 3: palliative n=4	100	100	0	4	n/a	15	Alopecia (in all patients), mild and consisted of	No	Prospective, case series, (n=5)	IV	1989 (accepted for publication)	Hussein et al. [28]

	infusion of 5-fluorouracil(5-FU) at a dose of 960 mg/m2/d for five days every 3 to 4 weeks.										mucositis, nausea, vomiting, reversible creatininemia, and transient azotemia. No dose modification was needed in any patient.					
	5-fluorouracil at 1,000 mg/m² /day 24-hour infusion for five days, in association with cisplatin at a dose of 100 mg/m²	No. of courses : 2,2,4,5		Adjuvant/Palliative; Jackson Stage: 3/4	25	25	0	n/a	n/a	7	well tolerated without severe toxicity or dose reduction.	No	Retrospective, single-center, (n=4)	IV	1980-1992	Kattan et al. [120]
	100 mg./m.2 cisplatin intravenously on day 1 and a 24-hour	38,5% received more than 2 cycles.		Neoadjuvant/Palliative; 25% Jackson stage III	25	25	0	n/a	n/a	responders: >32 and >57 non-	100%: nausea and vomiting. 37,5%:	No	Retrospective, single-center, (n=8)	IV	1985-1990	Shammas et al. [29]

	infusion of 1,000 mg./m.2 5-fluorouracil on days 1 to 5. The cycle was repeated every 3 to 4 weeks. The aim was to give at least 2 cycles.	Deterioration in general condition was the main reason for discontinuing chemotherapy after 1 or 2 cycles.		75% Jackson stage IV						responders: median: 9,5	poor chemotherapy tolerance. 37,5%: impairment of renal function. 25%: severe infection. 12,5%: tinnitus.					
VBM (Vincristine/Bleomycin/Methotrexate)	Day 1 Vincristine 0,5 mg/m2 Day 2 Bleomycin 7,5 mg/m2 and Methotrexate 12,5 mg/m2 Day 3 Bleomycin 7,5 mg/m2 and Methotrexate 12,5 mg/m2 Between two courses there was a 4-	The total number of courses per patient varied between 4 and 7.	No	Adjuvant; T2,3 N2 M1	n/a	n/a	n/a	n/a	n/a	well differentiated (50%): 27 poorly differentiated (50%): 41,5	Pyrexia, pneumonitis, fibrotic changes in both lungs (due perhaps to bleomycin)	Yes, radiotherapy	Retrospective, Case series, (n=4)	IV	1983 (accepted for publication)	Maiche et al. [19]

	week interval.															
	Bleo/Vin/MTX (Bleo 15 mg iv days 1 and 2, Vin 1 mg iv day 1, MTX 30–50 mg iv day 3, repeated with a 1-wk interval until maximum of 12 cycles)	n/a		Neoadjuvant; irresectable disease: T1-4 N0,1,3, M0	60	n/a	n/a	n/a	n/a	n/a	n=1 toxic-related death, autopsy-confirmed bleomycin pneumonia with lung emboli, 3 mo after start of chemotherapy;	No	Retrospective, single-institution, (n=5)	IV	1972-2005	Leijte et al. [52]
	Adjuvant/Neoadjuvant: vincristine 1 mg i.v. on day 1, bleomycin 15 mg i.m. 6 and 24 h after vincristine, methotrexate 30 mg orally on day 3. This	Yes		Adjuvant: 2-4 weeks after radical inguinal lymphadenectomy. Neoadjuvant: N3	Neoadjuvant: 60	Neoadjuvant: 60	Neoadjuvant: 0	n/a	Adjuvant: 91,7%: >42 (free of disease at follow up); 8,3%: relapse at 16 Neoadjuvant: 60% at	unreached	Myelosuppression, Lung fibrosis, Hyperpyrexia, Skin hyperpigmentation, Stomatitis.	No	Retrospective, Case series: Adjuvant (n=12) Neoadjuvant (n=5)	IV	1979-1985	Pizzocaro et al. [25]

	regimen was repeated weekly for a total of 12 weeks.								20,27,72 follow-up free of disease; 40% at 1,4 dead of disease		No toxic-related deaths were noted.					
PVF (Vincristine/Cisplatin/5-fluorouracil)	vincristine 1,2 mg/m2 i.v. day 1 , cisplatin 20 mg/m2 i.v. days 1 to 4, 5-fluorouracil 1 g/m2 i.v. day 5.	n=2: 2 cycles; n=1 n/a	No	Palliative; recurrent disease/ not complete resection	0	0	0	n/a	2	n/a	n/a	No	Retrospective, Case series, (n=3)			
Vincristine/Bleomycin	On day 1 of each week the patients were given 0,025 mg. per kilogram vincristine administered via rapid intravenous injection. Twelve hours later 15 mg. of bleomycin was given	total dose of 4, 12, 15 mg. of vincristine and 60, 225, 225 mg. of bleomycin.	No	Palliative; Metastatic	0	0	0	0	0	3	bleomycin pulmonary toxicity, pyrexia, stomatitis, paresthesia.	No	Retrospective, Case series, (n=3)	IV	1973	Williams et al. [23]

	intravenously. On day 4 of each week, each patient received an additional 15 mg. of intravenous bleomycin.															
BMP/CMB (Cisplatin/Methotrexate/Bleomycin)	75 mg/m2 cisplatin infused intravenously at a rate of 1 mg. per minute on day 1 and intravenous bolus of 25 mg./m2 methotrexate on days 1 and 8, and intravenous bolus of 10 units per m2 bleomycin on days 1 and 8. Chemotherapy was repeated every 21 days. Two cycles of chemotherapy	Median duration of treatment was 10 weeks (maximum 60). 15% completed treatment. After achieve CR chemotherapy was discontinued after 6 cycles.	No	Palliative; locally advanced or metastatic	32,5	20	12,5	n/a	~3,2	~6,4	100% treatment-related toxicity; G4: leukopenia, lymphopenia, thrombocytopenia, pulmonary embolism, anemia, granulocytopenia, prothrombin time of 100, elevated serum	No	multi-institutional (31); Phase II (n=40)	II	1986-1994	Haas et al. [27]

	constituted an adequate trial.										creatinine, infection. The most common toxicities were gastrointestinal (82,5%), hematological (67,5%) and mucosal (35%). 22,5%: incompleted therapy due to toxicity. 12,5%: treatment-related deaths (infection, pulmonary complication,					

											pneumonitis)					
	Cisplatin: 30 mg/m2 on days 1, 2 and 3; Methotrexate: 25 mg/m2 on days 1 and 8; Bleomycin: 15 mg on days 2, 5, 9 and 12; regimen was repeated after 3 weeks.	Median of 2 cycles (range 1-4 cycles).		Neoadjuvant; T1-3 N3 M0	62,5	62,5	0	n/a	n/a	The overall: 1-year: 70,8%, 2 -year: 50%, 5-year: 45,8% Responders:1-year: 86,7 %, 2-year: 73,3 %, 5-year: 73,3 % Non-responders:1-year: 44,4%, 2-year 11,1%, 5-year: 0 %	83,3%: G1-3 bone marrow suppression, 45,8%: G1-2 oral mucous damage. No toxic-related deaths.	No	Retrospective, single-center, (n=24)	IV	2001-2010	Zou et al. [198]
	Cisplatinum 20 mg/m2 i.v. on days 2–6, Methotrexate 200 mg/m2 on days 1, 15	median of 3,5 (3,2–6) courses per patient. (in all, 45		Adjuvant (n=8) and Palliative (n=5), pT1-4pN1-3M0, pT1-4 pN0-3M1,	Palliative: 0	Palliative: 0	Palliative: 0	n/a	Adjuvant: 37,5% patients: no evidence of disease	Adjuvant: 50% patients: 11; 37,5% patients:	G3-4: anaemia, leukocytopenia; G2-4:	No	Retrospective, single-center, (n=13)	IV	1996-2003	Hakenberg et al. [107]

	and 22 (= 1), Bleomycin 10 mg/m2 i.v. bolus on days 2–6, Cycle length of 21 days-chemotherapy was repeated every 21 days.	treatment courses were given); (All Adjuvantly-treated received 3 courses).		Systemic recurrence					at a median follow-up of 54 Palliative: 100% initially stable disease under treatment	5-year: 100% Palliative: 5	thrombocytopenia; Deep venous thrombosis, Stomatitis, Epistaxis, Lymphatic oedema, Paravasation, Pulmonary emboli, Interstitial pneumonia. One dead from restrictive pulmonary disease after a total dose of bleomycin of 250 mg.					

	200 mg/m² of methotrexate on days 1, 15, and 22, twenty-four-hour infusion of 10 mg/m² of bleomycin daily for five days, and 20 mg/m² /day of cisplatin for five days.	No. of courses : 3,4,4,4.		Adjuvant/Palliative; Jackson Stage 4	25	0	cCR: 25	n/a	n/a	7,5	well tolerated without severe toxicity or dose reduction.	No	Retrospective, single-center, (n=4)	IV	1980-1992	Kattan et al. [120]
	n=11 intravenously: 200 mg/m² of methotrexate on days 1, 15, and 22, 24-hour infusion of 10 mg/m² of bleomycin daily for 5 days, and on day 2 20 mg/m² /day of cisplatin infused during 1 hour daily for 5 days. n=3 intravenously: 200	20 intraarterial and 49 intravenous courses were given.		Palliative; Jackson Stage 4	72	57	cCR: 14	5,9	n/a	10	35,7%: evidence of bleomycin toxicity, increase in serum creatinine; mucositis, peripheral neuropathy	No	Retrospective, (n=14 included n=12 PSCC)	IV	1987-1989	Dexeus et al. [26]

	mg/m² of methotrexate on days 1, 15, and 22, intraarterially: cisplatin 100 mg/m² during 6 hours and 20 mg/m² bleomycin in 24-hour infusion for 2 days.															
	Bleo/Cis/MTX (Bleo 15 mg iv days 2–5, Cis 20 mg/m2 iv days 2–5, MTX 200 mg/m2 iv day 1, repeated with a 3-wk interval until maximum of 4 cycles)	n/a		Neoadjuvant; irresectable disease: T1-4 N0,1,3, M0	67	n/a	n/a	n/a	n/a	n/a	n=3: toxicity-related death within 2 wk after starting chemotherapy; tromboembolic complications involving brain stem infarction, within 2 wk after the start of	No	Retrospective, single-institution, (n=10)	IV	1972-2005	Leijte et al. [52]

											chemotherapy; bacterial pneumonia during a period of prolonged leucopenia, 3 mo after the start of chemotherapy; n=1: the discontinuation of treatment: G3 severe pulmonary toxicity, probably caused by bleomycin.					
	On days 1, 15 and 22, 200 mg/m2 methotrexate were	Yes		Neoadjuvant; T1,2,3N3M0 ,1	0	0	0	n/a	n/a	7	G3: 1 episode of bleomycin toxicity	No	Retrospective (n=3)	IV	1985-2000	Bermejo et al. [31]

	administered intravenously. On days 2 through 6 patients were given 10 mg/m2 bleomycin daily by intravenous infusion for a total of 50 mg/m2 as well as 20 to 30 mg/m2 cisplatin intravenously daily for a total of 5 days. Cycles were administered during 28 days for a total of 2 to 9 cycles.															
	200 mg./m.2 methotrexate on days 1, 15 and 22, and 20 mg/m.2 cisplatin and 10 mg./m.2 bleomycin on days 2	n/a		Palliative; Locally advanced or metastatic	57,1	38,1	cCR: 19	n/a	n/a	11,5 (72,4% with PSCC)	Combined with others genitourinary cancers	n/a	Phase II study; (n=21)	III	1988-1994	Corral et al. [199]

	through 6 during a 28-day cycle.															
	intra-arterial: Cisplatin 150 mg/m2 on days 1-6, and Methotrexate 200 mg/m2 on day 1, and Bleomycin 30 mg/m2 on day 1.	Adjuvantly: 3 and 4 cycles; Neoadjuvantly: 2 cycles		Adjuvant/Neoadjuvant T2N3M0, stage IV	100	Adjuvant: 100	Neoadjuvant: pCR: 100	Neoadjuvant: 8	n/a	Neoadjuvant: 22 Adjuvant: 5 and unreached	n/a	Yes, radiation therapy in 1 adjuvantly-treated patient	retrospective, case report, (n=3)	IV	1994-1999	Huang et al. [129]
	n/a	1-4 cycles		Second line; TxN2-3M0: progressive/recurrent disease	40	20	cCR: 20	n/a	n/a	4	20%: fatal pneumonitis	Yes, 20% gefitinib, 20% cetuximab/cisplatin	Retrospective, single-institution, (n=5)	IV	2000-2008	Wang et al. [200]
Methotrexate/Mitomycin C/Bleomycin/Cisplatin/5-fluorouracil	continuously pump-infused intraarterially for 2 days in each course: methotrexate (110 mg/m2 per day), mitomycin C (4,5 mg m2 per day),	median of 2 cycles (1-5)	No	Neoadjuvant; T1-3 N0-2 M0	overall: 83 inguinal node: palpable: 60 non-palpable: 100	50	cCR: 33,3	n/a	node-negative >60 node-positive <20	n/a	G1: Fatigue G1-2: Anorexia G3: Anaemia,Febrile neutropenia no toxic death	No	Retrospective, single-institution, (n=12)	IV	2005-2013	Chiang et al. [108]

	bleomycin (15 mg/m2 per day), cisplatin (35 mg m2 per day), and 5-fluorouracil (1200 mg/m2 per day) The course was repeated with an interval of 4 weeks.										was noted					
	infused continuously intraarterially for 2 days in each course: methotrexate (110 mg/m2 /day), mitomycin C (4,5 mg/m 2 /day), bleomycin (15 mg/m2/day), cisplatin (35 mg/m2/day) and 5-fluorouracil (1200 mg/m2/day). n=1: carboplatin (100	median of 3 cycles (3-5)		Neoadjuvant; T1-3 N0-2 M0	100	80	cCR: 20 (after 5 courses)	n=1: 4 n=2: 8	n/a	n=2: >13 and >15 recurrence n=2: 6,15	G1-2: anorexia G3: anaemia G3 febrile neutropenia	Yes, salvage radiotherapy in recurrence (n=2)	Retrospective, single-institution, (n=5)	IV	2005-2009	Chen et al. [118]

	mg/m2/day) was used instead of cisplatin and the doses of other regimens were adjusted to be 75% of regular dose (due to renal insufficiency) The interval of each course was 4 weeks.															
Bleomycin	Bleomycin: at doses of 3 to 20 mg/m2 i.v. in a continuous infusion and 10 to 30 mg/m2 in a daily or weekly bolus until mucositis was noted.	n/a	No	Adjuvant; local-regional and distant metastatic disease	21	14	cCR: 7	Median response duration: 3 (2-4)	n/a	n/a	mucositis, drug-related fever, drug rash, histological and/or clinical evidence of pulmonary toxicity, elevated liver function	No	Prospective; (n=14)	III	1984 (published)	Ahmed et al. [21,203]

											tests, alopecia					
	bleomycin: i.v. or i.m. the total dose ranged of 89- 212 mg/m2	continued after the total course of radiotherapy in patients with distant metastases.		Adjuvant; T1-2 N0,1 M0 or T2-3 N2-3 M1	n/a	n/a	n/a	without distant metastases: well differentiated: 85 poorly differentiated: 120	n/a	with distant metastases: well differentiated: 16 (33% >61) poorly differentiated: 10,5	Pyrexia, pneumonitis, fibrotic changes in both lungs (due perhaps to bleomycin)	Yes, radiotherapy	Retrospective; Case series, (n=10: n=3 without distant metastases, n=7 with distant metastases)	IV	1983 (accepted for publication)	Maiche et al. [19]
	1. bleomycin: 30 mg., i.v., twice a week (total dose, 460 mg.) and 60 mg., i.v., twice a week (total dose, 420 mg.) and 10 mg., daily (total dose,120 mg.). A total of 1000 mg. bleomycin was given in	n=1 after 150 mg still during therapy; n=1 the tumor disappeared after 255 mg n=1 after 12 injections of 15 mg		Adjuvant/Palliative; n/a	75	50	cCR: 25	n/a	n/a	6, >14, >7 and n/a	nausea, stomatitis, loss of appetite, pyrexia, pain along the vein of injection, induration of the vessel, sclerosis,	No	Retrospective, single-centre, case series, (n=8)	IV	1965-1968	Ichikawa et al. [15]

	127 days.<br><br>2. bleomycin: 15 mg., i.v., twice a week (total dose, 300 mg.) and 15 mg., i.m., twice a week (total dose, 290 mg.) and 300 mg., i.v. and 10 divided doses and 15 mg., i.v., twice a week (total dose, 120 mg.).<br><br>3. bleomycin: 15 mg., i.v. , twice a week (total dose, 690 mg.) and 15 mg., i.m., twice a week (total dose, 120 mg.);<br><br>4. bleomycin: 15 mg., i.m., twice a week, (total dose, 255	of bleomycin the tumor had almost disappeared. and n/a									pigmentation and hypersensitivity of the fingers and palms, alopecia, deformity of nails, pneumonia and pulmonary fibrosis, leukocytosis.<br><br>Treatment-related death: n=1: pneumonia					

	mg.)<br><br>5. bleomycin: 15 mg , i.v. once a week.<br><br>6. bleomycin: 15 mg i.v. twice a week.<br><br>7,8. bleomycin: n=1: a total of 1185 mg; n=1: a total of 580 mg															
	Bleomycin: 15 mg i.v. days 1 and 3, repeated weekly until total maximum dose of 200–300 mg)	n/a		Neoadjuvant; irresectable disease: T1-4 N0,1,3, M0	33	n/a	n/a	n/a	n/a	n/a	n/a	No	Retrospective, single-institution, (n=3)	IV	1972-2005	Leijte et al. [52]
Cisplatin	Cisplatin: 3 mg/kg or 120 mg/m2 Q 3-4W or 1,6-2,0 mg/kg Q 3W	an adequate trial was defined as 1 dose, and a 4 week survival	No	Palliative; Jackson stage III or IV	50	33	cCR: 17	7	n/a	n/a	universal: nausea, vomiting and anorexia; n=1: tinnitus, renal	No	Retrospective, single-centre, case series, (n=6)	IV	1979 (published)	Sklaroff et al. [20]

											dysfunction					
	Cisplatin: 50 mg/m2 i.v. in 1 to 4-hour infusion on days 1 and 8 of each cycle. Cycles were repeated every 28 days.	One cycle was considered an adequate trial.		Palliative; Jackson stage III or IV	15,4	15,4	0	Median response duration: 2 (1-3)	n/a	4,7	38%: moderate nausea/vomiting/anorexia; minimal leukopenia and thrombocytopenia, nephrotoxicity, hyperuricemia and anaemia.	No	Phase II; (n=26)	II	1988 (accepted for publication)	Gagliano et al. [202]
	Cisplatin: a dose of 70 or 120 mg/m2 i.v. every 3 weeks	n/a		Adjuvant; local-regional and distant metastatic disease	25	16	cCR: 8	Median response duration: 8 (2-8)	n/a	n/a	nausea and vomiting, ototoxicity, transient increase in the serum creatinine level	No	Prospective; (n=12)	III	1984 (published)	Ahmed et al. [203]

	Methotrexate: administered every 2 to 4 weeks at a dose of 250 mg/m2 i.v. In the absence of severe (G3/4) toxicity the dose was increased to 1,500 mg/m2 Patients attaining a complete or partial response thereafter were given a low dose of methotrexate (30 to 40 mg/m2 i.v. every week). n=8 received 30 to 40 mg/m2 i.v. every week for induction and maintenance therapy.	n/a		Adjuvant; local-regional and distant metastatic disease	61	53	cCR: 7	Median response duration: 3 (2-31)	n/a	n/a	mucositis, skin rash, reversible hepatic and pulmonary toxicity, reversible renal insufficiency, leukopenia, 1 treatment-related death of sepsis	No	Prospective; (n=13)			
Methotrexate	Methotrexate: high dose intravenous (3	After 10 weekly	No	Palliative; Metastatic	100	0	cCR: 100	complete clinical	>9	n/a	none	No	Retrospective, case-	IV	1976	Garnick et al. [16]

	gm/m2. Total dose of 5,8 gm.)	treatments placed on maintenance therapy, every 2 weeks with a total of 25 courses.						remission					report, (n=1)			
	Methotrexate: infused intraarterially continuously with 50 mg every 24 h using a portable pump	n/a		Neoadjuvant; T1,2,4 N0,1,2 Any stage	88	44	cCR: 44	n/a	n/a	complete responders: 16,9 years (7 mo-25,4 years)	G3,4 leucopenia, elevated transaminases, skin rash, thrombocytopenia, malaise, anorexia, nausea, fever, and diarrhea. The reasons for	No	Retrospective; single-center, (n=18)	III	1985-2009	Sheen et al. [207]

											stopping continuous MTX infusion: skin rash, thrombocytopenia, elevated transaminases, leucopenia, wound infection and malaise					
	methotrexate: 62,5%: intravenous high-dose methotrexate, 250-1500 mg/m2 Q 2-4 weeks (approximately 10-14 day intervals), 25%: intravenous low-dose methotrexate, 0,5-	high-dose methotrexate: 25%: 2 doses; 37,5%: 4 doses.		Palliative; Jackson stage III or IV	37,5	37,5 high-dose: 25% low-dose: 12,5%	0	3	n/a	n/a	mucositis, myelosuppression, dermatitis, gastrointestinal hemorrhage	No	Retrospective; case series; (n=8)	IV	1979 (accepted for publication)	Sklaroff et al. [18]

	3,0 mg/kg weekly; 12,5%: 5 mg orally methotrexate for 8 consecutive days.															
Cisplatin/Methotrexate	Methotrexate in association with Cisplatin at a dose of 100 mg/m²	No. of courses : 2,2,3	No			0	0			5		1 of 3 patients received radiotherapy. Excluded from results.	Retrospective, single-center, (n=3)			
Cisplatin/Adriamycin	Adriamycin in association with Cisplatin at a dose of 100 mg/m²	2 cycles complete	No			0	0			1		No	Retrospective, single-center, (n=1)			
Cisplatin/Bleomycin/Vinblastine	Bleomycin and Vinblastine in association with Cisplatin at a dose of 100 mg/m²	2 cycles complete	No	Adjuvant/Palliative; Jackson Stage: 4		0	0			3		No	Retrospective, single-center, (n=1)			
Cisplatin/Bleomycin/Epirubicin	Epirubicin and Bleomycin in association with Cisplatin at	2 cycles complete	No	Adjuvant/Palliative; Jackson Stage: 2	0	0	0	n/a	n/a	26	well tolerated without severe toxicity or dose reduction.	No	Retrospective, single-center, (n=1)	IV	1980-1992	Kattan et al. [120]

	a dose of 100 mg/m^2														
Epirubicin	Escalating doses of epirubicin (100-120 mg/m2) i.v. at 3-week intervals.	3 months duration	No	Second-line; Metastatic	100	0	cCR: 100	unreached	unreached	n/a	No renal or hepatic toxicity was encountered, no developed congestive cardiac failure.	No, but previously received methotrexate, cisplatin and etoposide combined chemotherapy.	Prospective; (n=1)	III	1989 (published)	Hickish et al. [130]
Adriamycin	Adriamycin intra-arterial at dose of 10 mg/m2 weekly for 3 months.	Yes	No	Palliative; T2N3M0, stage IV	0	0	0	stable disease	n/a	>3	n/a	No	retrospective, case report, (n=1)	IV	1994-1999	Huang et al. [129]
Peplomycin	Peplomycin was administered intravenously over 24 h at 10 mg twice a week, to a total dose of 60–70 mg.	Yes	No	Multidisciplinary treatment; well-differentiated, stage T2 tumour	100	0	pCR: 100	unreached	unreached	>7 years	no major side-effects related to the combined therapy	Yes, radiation was applied in 2-Gy fractions to the tumour area 5 days a week for 3	Prospective; (n=2)	III	1986-1991	Shirahama et al. [210]

												weeks at a total dose of 30 Gy and was followed twice a week by laser hyperthermia 30–60 min later. Both concurrently to chemotherapy.				
	10 mg peplomycin once a week for 10 weeks intravenously.	A total of 10 doses.		Neoadjuvant; T2 N2 M0	0	0	0	n/a	n/a	n/a	n/a	No				
Pirarubicin	pirarubicin, 10 mg/m2, was administered once a week for 4 weeks intravenously.	A total of 4 doses.	No	Neoadjuvant, second-line; T2 N2 M0	100	0	pCR:100	n/a	n/a	evaluated at 12 with no evidence of disease	n/a	Yes, radiotherapy: Linac radiotherapy between 40 Gy/4 weeks/2	Retrospective, case report, (n=1)	IV	1994	Igarashi et al. [209]

												0 fractions.				
