# Supplementary material for: Systemic Chemotherapy in Penile Squamous Cell Carcinoma: Mechanisms, Clinical Applications, and Evidence-Based Regimens
Source: Cancers (Basel). 2025 Dec 23;18(1):46. doi: 10.3390/cancers18010046 (PMC12785095; doi:10.3390/cancers18010046)
Supplement: Supplementary file 1 [file cancers-18-00046-s001.zip › Table S3.pdf]

Table S3: Evidence summary of chemotherapy regimens in PSCC—emerging, second-line, and other regimens																
**Regimen**	**The treatment regimen**	**Completion of Planned Cycles**	**Is in 2023 EAU-ASCO Collaborative Guidelines [8] preferred approach?**	**Indication, staging or TNM classification system**	**Objective Respons e Rate (ORR), (%)**	**Partial Respons e Rate (PRR), (%)**	**Pathological/Clinical complete response pCR/cCR (%)**	**Median time to progression (TTP), (months)**	**The median progression-free survival (PFS), (months)**	**the median overall survival (OS), (months)**	**Toxicity. (If Available: Common Terminology Criteria for Adverse Events (CTCAE) [46]; (G= Grade)**	**If simultaneous with other non-surgical therapy?**	**Study Design, (patients evaluable for response)**	**Level of Evidence the OCEBM criteria [47]**	**Patient Enrollment Period (years)**	**References**
Vinflunine	4 cycles of vinflunine 320 mg/m2 given on day 1 via intravenous infusion over 20 min given every 21 days.	55% completed at least 4 cycles. Dose reduction or delay was required for 20% of doses.	Yes, (palliative for platinum-unfit patients)	Palliative; Platinum-unfit/inoperable any T with N2/N3 or T4 any N.	27,3	27,3	0	n/a	2,9 1-year PFS: 16,7%	8,4 1-year OS: 33,7%	The adverse events leading to discontinuation: syndrome of inappropriate antidiuretic hormone secretion and pulmonary	No	single-arm, Phase II (n=22)	II	2014-2017	Nicholson et al. VinCaP Trial [42]

											embolus; constipation; neutropenia, pyrexia, and acute kidney injury.<br><br>The most common adverse events in any G: fatigue, constipation, decreased appetite, anaemia. The most common adverse event: G ≥3: Neutropenia 68% had at least 1 G≥3					

											adverse event.					
ITP (Docetaxel/Cisplatin/Ifosfamide)	up to 2–4 cycles every 21 days with docetaxel 75 mg/m2 on day 1, cisplatin 25 mg/m2 on days 1 to 3 and ifosfamide 1200 mg/m2 on days 1 to 3.	21% completed 4 cycles. 15,8 % completed 1 cycle because of disease progression (n = 2) and toxicity (n = 1). 63,2% completed 2 cycles and because of the good therapeutic effect, operations were carried out	No	Neoadjuvant, TxN3M0	63,2	63,2	0	n/a	11	23	G1-4: Myelosuppression G1-3:Nausea/vomiting G2: Allergic reaction G1-2: Myocardial ischemia, Alopecia, Motor neuropathy; There were no deaths related to the treatment protocol. n=1 discontinued chemotherapy due to severe	No	Retrospective, single-institution, (n=19)	IV	2009-2016	Xu et al. [182]

											myelo-suppression.				
Cisplatin/Irinotecan	Irinotecan on days 1, 8 and 15 of every 28-day cycle at a dose of 60 mg/m2 over 30 min in an i.v. infusion and cisplatin at a dose of 80 mg/m2 on day 1, after irinotecan, as a 1–3 h infusion. Neoadjuvant setting with a maximum of 4 cycles before surgery; Palliative setting up to 8 cycles.	Neoadjuvant: 4 (3–4) Palliative: 4 (1–8)	No	Neoadjuvant: T3 or N1-N2 Palliative: T4 or N3 or M1	30,8 Neoadjuvant: 28,6 Pallative: 31,6	Neoadjuvant: 14,3 Pallative: 26,3	cCR: Neoadjuvant: 14,3 Pallative: 5,3	n/a	n/a	n/a	Diarrhoea G3, n=3; Neutropenic fever G4, n=2; Pneumonia with G4 neutropenia, n=1 (stopped treatment); Others: WBC: G1-G4, ANC: G1-G4, Thrombocytopenia: G1,2,4, Haemoglobin: G1-G4, Hypercalcaemia: G1,2,4, Hypocalcae	No	non-randomised, prospective, Phase II, multicentre (6); (n=26)	II	2004-2006	Theodore et al. EORTC PROTOCOL 30992 [191]

											mia: G1,2,4, Hypotension: G1,3,4, Cardiac (thrombosis): G3,4; Fatigue: G1-G4, Neutropenic infection: G1-G4, Pulmonary (other G1,3, embolus: G4), Haemorrhage: G4, Diarrhoea: G1-G3					
Cisplatin/Gemcitabine	cisplatin (80mg/m2 on day 1) and gemcitabine (1,250mg/m2 on days 1 and 8) every 21 days.	6 cycles	No	Palliative; M1	100	100	0	12 (one patient)	n/a	n=1 stable disease >11 mo	n=1 Tinnitus (carboplatin was substituted for cisplatin after the	No	Retrospective; Case series, (n=2)	IV	2007 (accepted for publication)	Power et al. [94]

											fourth cycle)					
	intraarterial infusions of 900 mg/m2 gemcitabine and 30 mg/m2 cisplatin over 15 to 20 min every 7 days. 3 weeks of treatment followed by 1 week of rest was defined as a cycle.	≥2 cycles (2-6)		Adjuvant/Palliative/Second-line for locally advanced or recurrent PSCC; Tx,1-3 N2,3 M0	83,3	58,3	cCR: 25	20	n/a	responders: 24 non-responders: 12,5	33,33%: unilateral lower limb edema with pain G3/4; 25%: leukocytopenia G2/3; 16,7%: myelosuppresion, nausea/vomiting G1/2, anorexia G1/2,anemia G1/2, painless lower limb edema; 8,3%: thrombocytopenia G3/4,	No	Retrospective; Case series, (n=12: n=5 locally advanced, n=7 recurrent.)	III	1999-2011	Liu et al. [104]

											abnormal renal function G1.					
	association of gemcitabine (1,250 mg/m2 on day 1 over 30 minutes) with cisplatin (50 mg/m2 on day 1 over 1 hour preceded) day 1 and day 15 of a 28-day cycle. Duration of the treatment was up to 6 cycles.	median of 5 cycles (1-6); 24% received 6 cycles-the maximum.		Neoadjuvant/Palliative/Second-line; Unresectable loco-regional lymph nodes and/or distant metastases at initial diagnosis or at relapse; any TNM	8	n/a	n/a	5,48	n/a	14,98 2-years OS rate: 39,32%	Neutropenia: G1/2 (36%), G3/4 (16%); Anemia: G1/2 (4%), G3/4 (12%); Thrombopenia: G3/4 (8%); Nausea/Vomiting: G3/4 (8%); Renal failure: G3/4 (4%); Dysesthesia: G3/4 (4%); Asthenia: G3/4 (4%)	No	open multicentric, non-randomized, phase II trial, (n=25)	II	2004-2010	Houédé et al. [201]

											Therapy discontinued in 24% because of G3/4 toxic effects.					
	Gemcitabine (1250 mg/m2 on day one) and Cisplatinum (50 mg/m2 on day one) every 14 days.	6 cycles		Neoadjuvant; N3 M0	0	0	0	0	0	n/a	well tolerated	No	Retrospective; Case report, (n=1)	IV	2016 (accepted for publication)	Lapierre et al. [103]
Gemcitabine	Gemcitabine: 100 mg/m2 on weeks 1 and 2, 200 mg/m2 on weeks 3 and 4, 300 mg/m2 on weeks 6 and 7.	Yes. One injection was postponed.	No	Palliative; N3 M0	100	0	pCR: 100	n/a	n/a	>7 years	One injection was postponed because of G3 thrombopenia which rapidly and spontaneously recovered. G3 skin	Yes, the radiation regimen to the planning target volume consisted in 59.4 Gy in 33 fractions		IV		

											reaction in the inguinal region, which was managed with topical treatment.	over 50 days.				
Paclitaxel	3-h intravenous infusion of 175 mg/m2 paclitaxel every 21 d.	A paclitaxel dose reduction of 20% was adopted in 16% of cycles due to G3 haematologic and nonhaematologic toxicities. Treatment was delayed in 7,4% cycles.	No	Second line; Metastatic disease	20	20	0	n/a	~2,5	~5,3 Responders: ~7,4	G1-4: Neutropenia, Thrombocytopenia G1-3: Anaemia, Alopecia, Oral mucositis, Nausea/vomiting, Peripheral neuropathy, Constipation, Diarrhoea	No	single-arm, phase 2, multicentre study (5), (n=25)	II	2004-2011	Di Lorenzo et al. [34]

		Median number was 3 cycles (range: 2–10). Responsive patients were treated until 6, 9, 10 cycles were completed.														
Methotrexate/Vinblastine/Peplomycin/Carboplatin	methotrexate (20 mg/mm2; day 1), vinblastine (4 mg/mm2; day 1), peplomycin (10 mg; day 1) and carboplatin (100 mg/mm2; day 2); weekly basis for 7 weeks.	Yes	No	Multidisciplinary treatment; penile tumor with extensive regional metastasis;	100	0	cCR: 100	unreached	unreached	>7 years without any regional recurrence or distant metastasis.	None major complications	Yes. Radiation was given to the lesion 5 days a week for 5 weeks at a total dose of 50 Gy and interstitial laser hyperthermia was	Retrospective, case-report, (n=1)	IV	2008 (published)	Shirahama et al. [119]

												produced with a Nd:YAG laser where the lesion was heated to 42–43 °C for 15 min twice a week for 5 weeks immediately following radiation and during chemotherapy.					
Mitomycin C	A 20-mg dose of MMC was administered intravenously, initially at weekly intervals.	Median no. of cycles: 6 (2–12)	No	Second-line for metastatic disease; pT1-3 cN1-3 cM0-1 G3	11	0	0	progressive disease	progressive disease, 11% stable disease in 18 mo evaluation	5	G3/4: thrombocytopenia and G2/3 leukopenia, anaemia	No	single arm, retrospective, monocentric, (n=9)	IV	2018-2022	Draeger et al. [212]

	After the second or third application of MMC, the treatment intervals were extended to 3 weeks. The application was continued until tumor progression or intolerable side effects occurred										occurred in all patients; G2/3: nausea and vomiting; G3: stomatitis					
Cisplatin/Capecitabine	n/a	n/a	No	Palliative, first-line; Metastatic	39	30	cCR: 9	n/a	n/a	n/a	G3/4: Fatigue, Neutropenia, Anaemia, Thrombocytopaenia, Oral mucositis, Diarrhoea, Hearing loss/tinnitus, Pneumonia,	No	Retrospective, single-institution, (n=33)	II	2006-2020	Liu et al. [204]

											Urosepsis, Renal failure, Palmer plantar erythrodysesthesia					
Oxaliplatin/Capecitabine	n/a	2 cycles	No	Palliative, second-line; Metastatic	0	0	0	n/a	n/a	n/a	n/a	No	Retrospective, case report, (n=1)	IV	2013 (published)	Pandey et al. [56]
Gemcitabine/Vinorelbine	n/a	4 cycles	No	Palliative, third-line; Metastatic	0	0	0	n/a	n/a	n/a	n/a	No				
	n/a	n/a		Second line; TxN2-3M0: progressive/recurrent disease	n/a	n/a	n/a	n/a	n/a	9,1	n/a	No	Retrospective, single-institution, (n=1)	IV	2000-2008	Wang et al. [200]
Gemcitabine/Docetaxel	n/a	n/a	No	Second line; TxN2-3M0:	n/a	n/a	n/a	n/a	n/a	8,5	n/a	No	Retrospective, single-institution, (n=1)	IV	2000-2008	

				progressive/recurrent disease												
Cabazitaxel	1-hour infusion of cabazitaxel at a dose of 25 mg/m2 every 21 days.	6 cycles were planned. A median of 2 cycles (range, 2–5) achieved.	No	Second line; M1; TxN3M0; inoperable TxN2M0; T4, any N, M0, progressive/recurrent disease	0	0	0	n/a	1,3	5,6	n=3: G3/4 anaemia, sepsis, and vomiting, n=4: neutropenic sepsis, hypercalcemia, sepsis, fever. no dose reductions or treatment delays were needed	No	Phase II, single-arm trial, (n= 9)	II	2014-2016	Challapalli et al. [214]
