# Supplementary material for: Systemic Chemotherapy in Penile Squamous Cell Carcinoma: Mechanisms, Clinical Applications, and Evidence-Based Regimens
Source: Cancers (Basel). 2025 Dec 23;18(1):46. doi: 10.3390/cancers18010046 (PMC12785095; doi:10.3390/cancers18010046)
Supplement: Supplementary file 1 [file cancers-18-00046-s001.zip › Text S1.pdf]

Text S1: Mechanistic Drug Classes – A Broader Context

S1.1. Mechanistic Drug Classes - Rationale

In order to ensure a detailed and comprehensive context of the understanding of chemotherapeutic agents used in penile squamous cell carcinoma (PSCC), we additionally outline their broader mechanisms of action and clinical applications beyond PSCC, thereby providing a broader scientific perspective.

S1.1.1. Alkylating Agents - Metal Salts

S1.1.1.1. Cisplatin

Cisplatin was first synthesized in 1845 by Michele Peyrone and became known as Peyrone's chloride. Its structure was later elucidated by Alfred Werner, forming a significant basis for coordination chemistry. In the 1960s, Barnett Rosenberg at Michigan State University discovered cisplatin's biological activity while investigating the effects of electric fields on bacterial growth, observing that platinum electrodes released a compound that inhibited cell division in Escherichia coli. Leading to the identification of cisplatin's biological activity. Following preclinical studies, cisplatin entered human clinical trials in 1971-72, with early success observed in treating testicular and ovarian cancers. Cisplatin was approved by the Food and Drug Administration (FDA) in 1978, revolutionizing cancer treatment [59-62].

The chemical structure of cisplatin consists of a platinum center bound to two chloride ligands and two ammonia ligands in a cis configuration. This configuration is crucial for its biological activity, as the trans isomer lacks anticancer properties. The mechanism of action of cisplatin begins with its activation inside the cell, where cisplatin undergoes hydrolysis, leading to the formation of covalent bonds with DNA, leading to the formation of intra- and inter-strand crosslinks, particularly at the N7 position of reactive center on purine residues preferentially of guanine. The 1,2-intrastrand cross-links of purine bases with cisplatin are the most notable among the changes in DNA. This DNA damage disrupts replication and transcription, triggering cell cycle arrest and apoptosis in rapidly dividing tumour cells. Although only a small fraction of intracellular cisplatin reaches the nucleus (1-10%), this is sufficient to initiate cytotoxic responses. However, to improve its efficacy, efforts have been made to identify synergistic mechanisms. Additionally, cisplatin-DNA adducts can be recognized by proteins such as high-mobility group (HMG) proteins and transcription factors like TBP, which may either shield the damage from repair or interfere with essential cellular processes, further promoting cell death. Other mechanisms contributing to cytotoxicity include inhibition of RNA polymerase II, telomere shortening, disruption of protein ubiquitination, and activation of the p53 pathway. These diverse pathways collectively enhance the antitumour efficacy of cisplatin [57,58,65,70].

Cisplatin has a broad spectrum of activity and is used in the treatment of multiple cancer types. In testicular cancer, the standard regimen for non-seminomatous germ cell tumour (NSGCT) is BEP (bleomycin, etoposide, cisplatin) therapy. In bladder cancer, it is frequently included in treatment protocols, especially for muscle-invasive cases. Cisplatin is also used in other cancers such as non-small-cell lung carcinoma (NSCLC), ovarian, and head and neck cancers, typically in combination regimens or alongside radiotherapy to enhance treatment efficacy [63,64,71,72]. Despite its efficacy, cisplatin's clinical use is sometimes limited by its toxicity profile. Nephrotoxicity, ototoxicity, and neurotoxicity remain significant concerns, driving the search for protective agents and alternative dosing strategies [66-68] also, cisplatin intolerance, even reported by Sekine et al. in penile cancer treatment [69].

S1.1.1.2. Carboplatin

Carboplatin was developed in the late 1970s as a safer, second-generation analogue of cisplatin, based on the hypothesis that a more stable leaving group than chloride could reduce toxicity without compromising antitumour efficacy. This led to the selection of JM8 (carboplatin) at the Institute of Cancer Research, among hundreds of screened platinum complexes. The FDA approved carboplatin in 1989 for the treatment of ovarian cancer [73,74].

Carboplatin is a platinum-based chemotherapeutic agent developed as a second-generation analogue of cisplatin. Similarly to cisplatin, its primary mechanism of action involves the formation of platinum-DNA adducts, which disrupt DNA replication and transcription, ultimately triggering apoptosis. While carboplatin retains the core platinum structure responsible for antitumour activity, it features a key chemical modification: a bidentate cyclobutane dicarboxylate ligand. This structural change significantly alters its pharmacokinetics by slowing the rate of aquation and DNA binding. As a result, carboplatin exhibits reduced reactivity and lower acute toxicity, particularly in terms of nephrotoxicity and neurotoxicity, compared to cisplatin. Although higher concentrations are often required to achieve therapeutic efficacy, carboplatin delivers comparable clinical outcomes, especially in the treatment of ovarian cancer, with improved tolerability and a more favourable safety profile [70,75].

Carboplatin is widely used in oncology due to its broad activity and improved tolerability compared to cisplatin. It is a standard treatment for epithelial ovarian cancer, often combined with paclitaxel [76]. In NSCLC and small cell lung cancers (SCLC), carboplatin-based regimens offer effective alternatives, particularly when cisplatin toxicity is a concern. It is also employed in head and neck cancers as a radiosensitizer and in patients with contraindications to cisplatin. Additionally, carboplatin is used in germ cell, bladder, and various other solid tumours, especially when a more favourable toxicity profile is needed [71]. Compared to cisplatin, carboplatin showed significantly reduced nephrotoxicity, gastrointestinal, and neurotoxic effects. However, its dose-limiting toxicity is myelosuppression, mainly thrombocytopenia [74].

S1.1.2. Alkylating Agents - Mustard Gas Derivatives

S1.1.2.1. Ifosfamide

A synthetic alkylating agent belonging to the oxazaphosphorine class and is structurally related to cyclophosphamide. The history of ifosfamide traces back to early 20th-century observations of mustard gas toxicity during World War I, which laid the groundwork for the development of alkylating agents in cancer therapy. Scientific efforts in chemical carcinogenesis, initiated in London and later advanced in Yale and Chicago, ultimately culminated in pharmaceutical innovation in Bielefeld, Germany. These efforts led to the development of oxazaphosphorine derivatives, with cyclophosphamide introduced in 1958, followed shortly by its structural isomer, ifosfamide, in the early 1970s. Designed to broaden the antitumour spectrum beyond that of cyclophosphamide, ifosfamide became a vital chemotherapeutic agent with FDA approval in 1988 [77,78].

Ifosfamide exerts its cytotoxic and anticancer effects through DNA alkylation and cross-linking, which impair essential cellular processes. Ifosfamide is a prodrug that requires metabolic activation in the liver by cytochrome P450 enzymes (mainly CYP3A4), ifosfamide is converted into 4-hydroxy-ifosfamide and then aldoifosfamide, which decomposes into the active metabolite ifosforamide mustard. This metabolite forms covalent bonds with DNA bases primarily at the N-7 position of guanine, resulting in intra- and interstrand cross-links. These cross-links prevent proper DNA replication and transcription, triggering DNA damage responses that lead to apoptosis. The formation of

interstrand cross-links is especially cytotoxic, as they are more difficult to repair, making ifosfamide particularly effective against rapidly dividing cancer cells [79,85].

Ifosfamide has broad clinical applications. It is integral in treating relapsed or refractory testicular cancer [83], soft tissue and bone sarcomas [84], and both Hodgkin and non-Hodgkin lymphomas [81,135]. As well as in lung cancer, especially SCLC. It also demonstrates activity in advanced ovarian, and breast cancers, as well as paediatric solid tumours like Ewing's sarcoma, Wilms' tumour and rhabdomyosarcoma [85]. The use of ifosfamide must be balanced against its side effect profile, which includes myelosuppression, neurotoxicity, and urotoxicity, specifically haemorrhagic cystitis necessitating the co-administration of protective agents like Mesna [80,85].

S1.1.3. Antimetabolites - Pyrimidine Antagonists

S1.1.3.1. 5-Fluorouracil

5-FU was developed in 1957 by Heidelberger et al. during research into antimetabolites as potential anticancer agents [87]. Early studies showed that fluorinated pyrimidines could effectively disrupt nucleic acid metabolism in tumour cells [88]. 5-FU received FDA approval in 1962 as a chemotherapeutic agent [92].

5-FU is a pyrimidine antagonist, being a fluoropyrimidine antimetabolite, structurally the uracil analogue. It exerts its anticancer effects through inhibition of thymidylate synthase (TYMS) and incorporation into nucleic acids [93]. Intracellularly, 5-FU is metabolized into active forms, including FdUMP, which forms a stable ternary complex with TS and reduced folate, thereby blocking the synthesis of deoxythymidine monophosphate (dTMP), an essential precursor for DNA replication and repair induces a state of "thymine-less death" in cancer cells. Additionally, metabolites such as FUTP and FdUTP are incorporated into RNA and DNA, by mimicking the natural pyrimidine bases, particularly uracil respectively, disrupting RNA processing and causing DNA damage. These combined effects result in impaired nucleic acid function and ultimately lead to cancer cell death [86,89].

Over the years, 5-FU has become a key part in the treatment of various cancers. In colorectal cancer, it remains a mainstay in both adjuvant and palliative treatment regimens, frequently used in combination with agents such as oxaliplatin or irinotecan [88]. In breast cancer and head and neck cancers, 5-FU forms part of several combination therapies [90]. 5-FU is also widely employed in the management of other gastrointestinal cancers, including those of the stomach, pancreas, and oesophagus [91].

S1.1.3.2. Gemcitabine

Gemcitabine (2′,2′-difluoro-2′-deoxycytidine, dFdC) was originally synthesized as a potential antiviral compound, but daily in vivo administration revealed a narrow therapeutic index. A change to staggered dosing schedules uncovered its potent antitumour activity in murine and human xenograft models, shifting its development toward oncology [101]. Belonging to the antimetabolite class alongside 5-fluorouracil and methotrexate, gemcitabine soon demonstrated broad-spectrum efficacy against solid tumours. Following extensive clinical evaluation, the FDA approved gemcitabine in 1996 for the treatment of pancreatic adenocarcinoma, with later approvals for several other malignancies [100,102], establishing it as one of the most important cytidine analogues introduced since cytosine arabinoside [105].

Gemcitabine is a nucleoside analogue prodrug that requires intracellular phosphorylation by deoxycytidine kinase to generate its active metabolites. Once converted, gemcitabine diphosphate (dFdCDP) and triphosphate (dFdCTP) exert complementary and self-potentiating effects. dFdCTP incorporates into DNA in place of deoxycytidine triphosphate (dCTP), producing masked chain termination: following incorporation and the addition of one further nucleotide, DNA polymerase stalls, replication forks collapse, and

apoptosis is triggered. At the same time, dFdCDP is a potent inhibitor of ribonucleotide reductase, which depletes intracellular dNTP pools, particularly dCTP, thereby enhancing further dFdCTP incorporation into DNA. Also, gemcitabine metabolites inhibit dCMP deaminase and CTP synthetase, prolonging nucleotide retention inside cells, and making tumour cells unable to efficiently excise gemcitabine from DNA [95,101,105]. Beyond its cytotoxic effects, gemcitabine has been shown to influence tumour-immune interactions, including upregulation of molecules such as MHC class I and PD-L1, and selective depletion of myeloid-derived suppressor cells, which may enhance antitumour immune responses [100].

Gemcitabine is used across a wide range of solid tumours. It remains a first-line option, for advanced pancreatic adenocarcinoma, frequently paired with nab-paclitaxel and NALIRIFOX or FOLFIRINOX [99,102, 106]. It is effective in recurrent ovarian cancer, both platinum-sensitive and resistant, often in combination with carboplatin [97,99], and has shown activity in soft tissue sarcomas as first, second- or latter line [98,99]. It is also widely used in combination with platinum agents for advanced NSCLC and in metastatic breast cancer resistant to anthracyclines and taxanes [99]. In urology, it is administered intravesically for non-muscle invasive bladder cancer (NMIBC) and combined with cisplatin as standard therapy for muscle-invasive disease (MIBC) [95,96].

S1.1.4. Antimetabolites - Folic acid antagonists

S1.1.4.1. Methotrexate (MTX)

An antimetabolite chemotherapeutic agent and immunosuppressant originated from research on folic acid in 1945 by Yellapragada Subbarow, who discovered that certain folic acid derivatives could cause tumour regression by inhibiting DNA synthesis [117]. This led Farber et al. to test folic acid antagonists - aminopterin and later amethopterin (methotrexate) to treat childhood leukaemia, achieving the first temporary remissions in 1948 and marking the beginning of modern chemotherapy [116]. Due to aminopterin's instability, MTX became the preferred drug. MTX was approved by the FDA for psoriasis in 1972 and for rheumatoid arthritis in 1988 [114]. The growing awareness in the late 1940s and early 1950s of MTX's potential as a cancer chemotherapeutic agent further accelerated its development and clinical use [115].

Due to the focus of this review, we have presented the mechanism of action of MTX in the context of chemotherapeutic dosing. At high doses (up to grams), methotrexate functions primarily as an antimetabolite and cytotoxic agent. MTX is structurally similar to folic acid and acts as a potent inhibitor of folate-dependent enzymes, especially dihydrofolate reductase (DHFR). Once inside the cell, MTX is converted by folylpolyglutamyl synthase (FPGS) into methotrexate polyglutamates (MTXGlu), which have increased intracellular retention and greater inhibitory potency. By binding to DHFR, MTXGlu blocks the reduction of dihydrofolate to tetrahydrofolate, a critical step in folate metabolism. The subsequent depletion of tetrahydrofolate limits the synthesis of thymidylate and purine nucleotides, thereby impairing DNA replication and repair. This disruption primarily affects cells in the S-phase of the cell cycle, where DNA synthesis occurs, leading to cell cycle arrest and the initiation of apoptosis. Other folate-dependent enzymes inhibited by MTXGlu TYMS and AICAR transformylase (AICART), further depleting nucleotide pools and disrupting DNA replication and repair. These actions are particularly toxic to rapidly dividing cells, such as malignant cells in leukaemia, lymphoma, and certain solid tumours, leading to cell cycle arrest and apoptosis [109,110,114].

Methotrexate plays a crucial role in the treatment ALL, where it helps prevent central nervous system involvement through intrathecal administration. It is also a key component in combination regimens for non-Hodgkin lymphoma [111]. In solid tumours, high-dose methotrexate is used effectively against osteosarcoma, head and neck cancers, breast

cancer, and choriocarcinoma [112]. MTX remains a cornerstone of chemotherapy protocols for a broad range of cancers despite its toxicity in high doses to the gastrointestinal mucosa, neurotoxicity, and the most dangerous - myelosuppression, among others. To prevent these side effects, Leucovorin is strongly recommended [113].

S1.1.5. Antitumour Antibiotics – Anthracyclines

S1.1.5.1. Doxorubicin (Adriamycin)

Doxorubicin (Adriamycin) is a member of the anthracycline family of chemotherapeutic agents. Originally isolated in the 1960s from the soil bacterium Streptomyces peucetius var. caesius, doxorubicin was developed as a structural analogue of daunorubicin, which had demonstrated potent antitumour activity but limited therapeutic application due to high toxicity. Chemical modification of daunorubicin yielded doxorubicin, which exhibited broader efficacy across both hematologic and solid tumours and a more favourable therapeutic profile, rapidly positioning it as a cornerstone in oncology approved for clinical use by FDA in 1974 [121-123].

Doxorubicin acts through interconnected nuclear, mitochondrial, and immunological mechanisms. In the nucleus, it intercalates between DNA base pairs, inhibits topoisomerase II by stabilizing the cleavable complex, induces double-strand breaks, alters DNA topology, destabilizes nucleosomes, and at higher concentrations forms covalent DNA adducts at 5′-GpC-rich sites. Nuclear entry is facilitated by 26S proteasome binding, but DNA remains its primary target. In the cytoplasm and mitochondria, doxorubicin undergoes redox cycling to generate reactive oxygen species (ROS) directly and via iron complexes, driving lipid peroxidation, protein oxidation, and mitochondrial dysfunction. Binding to cardiolipin and inhibition of ETC complexes I/II further amplify ROS, while disruption of SERCA and ryanodine receptor function perturbs Ca^{2+} homeostasis, leading to apoptosis and necrosis. Doxorubicin also elevates ceramide levels, activating AMPK, inhibiting mTORC1, and inducing pro-apoptotic transcription, although resistance may arise through glucosylceramide synthesis. Beyond direct cytotoxicity, it promotes immunogenic cell death by exposing calreticulin and HSP70/90 on the tumour cell surface, releasing HMGB1, and stimulating IL-1 and type I IFN secretion, thereby enhancing dendritic cell maturation, antigen presentation, and NK- and T-cell–mediated clearance. These combined actions explain its broad efficacy but also its dose-limiting cardiotoxicity, driven by persistent ROS and Ca^{2+} dysregulation [121-123].

Doxorubicin is integral to numerous standard chemotherapy regimens across both hematologic and solid tumours [121-123]. In breast cancer, it is incorporated into AC (doxorubicin/cyclophosphamide) and FAC (5-fluorouracil/doxorubicin/cyclophosphamide) protocols, widely used in adjuvant and neoadjuvant settings [124,125]. In lymphomas, it forms part of CHOP (cyclophosphamide, doxorubicin, vincristine, prednisone) for non-Hodgkin lymphoma and ABVD (doxorubicin, bleomycin, vinblastine, dacarbazine) for Hodgkin lymphoma, both of which remain standards of care [126]. Used also in treatment of soft tissue sarcoma and osteosarcoma, often combined with ifosfamide or cisplatin [127,128]. Its use further extends to ovarian, bladder, gastric, thyroid, and other solid tumours [123], underscoring its broad clinical relevance despite the persistent challenge of cumulative cardiotoxicity.

S1.1.5.2. Epirubicin

First introduced in the late 1970s, epirubicin is an anthracycline chemotherapeutic agent developed as a stereoisomer of doxorubicin (adriamycin), differing only in the orientation of the 4′-hydroxyl group what results in greater lipophilicity, enhanced cellular uptake, and more efficient hepatic elimination through glucuronidation, which collectively contribute to reduced systemic and cardiac toxicity a conclusion reinforced at a 1984

international symposium in Milan. Since the 1980s, it has become a key component of combination chemotherapy regimens, particularly in breast cancer, bladder cancer, and lymphomas [131].

Epirubicin shares the fundamental mechanisms of anthracycline cytotoxicity with doxorubicin, including DNA intercalation and inhibition of topoisomerase II, which disrupt replication and transcription and lead to double-strand DNA breaks. Like doxorubicin, it can also generate reactive oxygen species through redox cycling, contributing to DNA damage and lipid peroxidation. However, the equatorial orientation of the 4′-hydroxyl group in epirubicin alters its pharmacological behaviour: it lowers the pKa, increases lipophilicity, and enhances passive cellular uptake, resulting in faster and higher intracellular accumulation than doxorubicin. At the same time, epirubicin undergoes efficient glucuronidation and more rapid elimination, leading to reduced systemic exposure. These differences translate into lower levels of ROS generation in cardiac tissue and substantially less cardiotoxicity compared with doxorubicin. Thus, while both agents act through similar cytotoxic pathways, epirubicin's stereochemistry and metabolism provide a more favourable therapeutic index [131,132].

Epirubicin is widely used in the treatment of diverse malignancies, most notably as part of combination chemotherapy regimens. In breast cancer in both adjuvant and neoadjuvant therapy, forming the basis of protocols such as FEC (5-fluorouracil, epirubicin, cyclophosphamide) and EC (epirubicin, cyclophosphamide). Its role extends to bladder cancer, where it is given intravesically for NMIBC. Epirubicin is also incorporated into regimens for lymphomas, including both Hodgkin's and non-Hodgkin's types, and has demonstrated efficacy in gastrointestinal malignancies such as gastric and oesophageal cancers. Beyond these primary indications, it has been explored in lung, ovarian, and sarcomatous tumours, reflecting its versatility and favourable therapeutic profile compared with doxorubicin [133-137].

S1.1.6. Antitumour Antibiotics – Miscellaneous

S1.1.6.1. Bleomycin

Bleomycin, first discovered in 1966, is a glycopeptide antibiotic derived from the soil bacterium Streptomyces verticillus [141,142]. It was approved by the FDA in 1973 for clinical use as a chemotherapeutic agent [138].

Bleomycin demonstrates its cytotoxic effects primarily through DNA strand breakage. It binds to DNA and, in the presence of iron and oxygen, generates reactive oxygen species (ROS) that induce single- and double-strand breaks. This damage disrupts DNA synthesis and triggers cell cycle arrest, particularly in the G2 phase. The accumulation of unrepaired DNA damage subsequently activates apoptotic pathways, leading to cell death. Bleomycin's selective toxicity toward actively dividing cells, especially those in the M and G2 phases, determines its effectiveness against certain cancers, although resistance mechanisms, such as hypoxia, metabolic inactivation, and enhanced DNA repair, can limit its efficacy [139,144]. Bleomycin plays a particularly important role in the BEP regimen for NSGCT [143,145] and the ABVD regimen (Adriamycin, Bleomycin, Vinblastine, and Dacarbazine) for Hodgkin's lymphoma [146].

Bleomycin has shown activity in various Germ-Cell Tumours [140] and squamous cell carcinomas, including those affecting the head and neck, cervix, and skin [147]. Although its clinical use is limited by pulmonary toxicity [8,138,146].

S1.1.6.2. Mitomycin C

Mitomycin C (MMC) is an antitumour antibiotic isolated in the 1958 from Streptomyces caespitosus and introduced into clinical use in the 1960s with FDA approval in 1974. As the best-studied mitomycin, it is the prototype of bio reductive alkylating agents, exerting cytotoxicity through DNA cross-linking under hypoxic conditions, a key

advantage in resistant tumour microenvironments. It shows broad activity against gastro-intestinal, genitourinary, and respiratory malignancies. Despite its therapeutic promise, cumulative hematologic toxicities such as thrombocytopenia and leukopenia, together with rare but severe complications including haemolytic–uremic syndrome, pulmonary toxicity, and cardiac failure, limited its widespread use. Despite these drawbacks, MMC's unique hypoxia-selective activity has preserved its clinical and investigational relevance for decades [148-150].

MMC is a bioreductive alkylating agent activated under hypoxic conditions commonly present in solid tumours. Following intracellular reduction by enzymes such as NADPH-cytochrome P450 reductase the resulting intermediates covalently bind DNA, generating interstrand cross-links mainly at guanine–cytosine (5′-CpG)–rich regions, which block replication and transcription and trigger apoptosis. DNA damage occurs throughout the cell cycle but is most pronounced in late G1 and early S phase, reflecting MMC's preference for heterochromatic, GC-rich regions that replicate late. In addition, MMC can generate DNA monoadducts and oxidative stress, further destabilizing the genome [148,150].

MMC has demonstrated efficacy across a wide range of malignancies, though its role has evolved with the advent of newer agents. In bladder cancer, intravesical MMC remains an effective cytotoxic option for superficial and carcinoma-in-situ disease; recent evidence shows it also significantly reduces recurrence in intermediate-risk non-muscle-invasive bladder cancer, with outcomes approaching those of BCG in non-CIS settings [151]. It has also been used in combination regimens such as FAM (5-FU/Doxorubicin/MMC) for gastric and pancreatic cancers, and in chemoradiation protocols for anal canal carcinoma. MMC demonstrated activity in breast cancer, often in combination with vinca alkaloids or as salvage therapy in anthracycline-pretreated patients, as well as in cervical, head and neck cancers and NSCLC where combinations like MVP (MMC/Vinblastine/Platinum) provided palliative benefit. However, the emergence of taxanes, platinum analogs, and targeted therapies has narrowed its use to select indications where its unique pharmacology, particularly hypoxia-directed activity and intravesical delivery clinical value [149].

S1.1.7. Plant Alkaloids - Vinca Alkaloids

S1.1.7.1. Vincristine

Vincristine is a first-generation vinca alkaloid derived from the Madagascar periwinkle (Catharanthus roseus) first isolated in 1961 [158]. Approved in 1963 by the FDA [157].

Vincristine works by binding to the β-subunit of tubulin at the interface between two tubulin heterodimers, causing the fragmentation and abnormal reassembly of microtubules into disordered, non-functional structures, thereby impairing mitosis by inhibiting microtubule polymerization, which is essential for cell division. This interruption of the cell cycle leads to metaphase arrest and subsequent apoptosis in rapidly dividing cells. Even at low doses, it interferes with microtubule dynamics by binding to their ends, further disrupting proper cell division. However, because microtubules also play a critical role in neuronal function, their disruption can have harmful effects on the nervous system. The most serious side effect of vincristine treatment is neurotoxicity, which primarily appears as vincristine-induced peripheral neuropathy (VIPN) [152].

Due to its significant efficacy, vincristine is a mainstay in the treatment of various hematologic malignancies and solid tumours. Following its discovery, vincristine was integrated into clinical practice, especially in paediatric oncology [153]. Its introduction revolutionized the treatment of acute lymphoblastic leukaemia (ALL) Hodgkin's, and non-Hodgkin's lymphoma [153-155,157]. It is also used in combination regimens for solid tumours like neuroblastoma, rhabdomyosarcoma, and Wilms' tumour [153,156].

S1.1.7.2. *Vinblastine*

Vinblastine is a first-generation vinca alkaloid chemotherapeutic agent widely used in the treatment of various malignancies, especially haematological ones. Vinblastine was developed in the 1950s, derived from the periwinkle plant (Catharanthus roseus) and was approved by FDA in 1963 [157,159,160,161].

Vinblastine acts by binding to the β-subunit of tubulin dimers, preventing their polymerization into microtubules that are essential for mitosis. This disruption blocks spindle formation, induces metaphase arrest, and leads to apoptotic cell death. While this core mechanism is shared across the vinca alkaloids, vinblastine is distinguished by several unique features. Structurally, it differs from vincristine by having a methyl group instead of a formaldehyde moiety on the vindoline subunit, a change that underlies its distinct pharmacological profile. Functionally, vinblastine is notable for its dose-limiting toxicity of bone marrow suppression, in contrast to vincristine's neurotoxicity. Moreover, research has shown that the C20′ position of vinblastine is very important for how strongly it binds tubulin and for its overall activity. Changing this site can improve potency and overcome resistance mediated by P-glycoprotein efflux, a feature specifically demonstrated for vinblastine and not generalized to other vinca alkaloids [157,161].

Vinblastine is clinically applied in the treatment of a broad range of malignancies, most prominently as a core component of the ABVD regimen (doxorubicin/bleomycin/vinblastine, dacarbazine) for Hodgkin's lymphoma, where it significantly improves outcomes. Likewise in non-Hodgkin's lymphoma, testicular germ cell tumours, and in advanced or refractory Kaposi's sarcoma, in the PVB regimen (vinblastine/bleomycin/cisplatin) [159,163]. Beyond above, vinblastine has demonstrated efficacy against several solid tumours, including breast cancer, bladder cancer, renal cell carcinoma and select sarcomas, typically as part of multi-agent chemotherapy [162].

S1.1.7.3. *Vinflunine*

Vinflunine was developed to improve upon earlier vinca alkaloids, such as vinblastine and vinorelbine, by increasing cytotoxic efficacy and reducing toxicity, approved for therapeutic use in Europe since 2008 [159,164,165].

Vinflunine, a bifluorinated derivative of vinorelbine, binds to the Vinca site on tubulin and suppresses microtubule dynamic instability and treadmilling at low concentrations, leading to G2/M arrest and apoptosis. At higher concentrations, it inhibits microtubule assembly, disrupts the interphase microtubule network, and induces paracrystal formation. Compared to vinblastine and vinorelbine, vinflunine has lower tubulin-binding affinity, does not suppress microtubule shortening, and inhibits treadmilling less potently features associated with reduced the risk of peripheral neuropathy and more reversible mitotic effects. It also exhibits antiangiogenic, vascular-disrupting, antimetastatic, and radiosensitizing properties, supporting its broad anticancer efficacy [165-167].

Vinflunine is approved as a second-line treatment for advanced or metastatic urothelial carcinoma in patients who have progressed after platinum-based chemotherapy [168,169]. It has also been explored in triple-negative and metastatic breast cancer [170], and in combination with cisplatin for NSCLC, showing promising results in phase II/III trials [171,172].

S1.1.8. *Plant Alkaloids – Taxanes*

S1.1.8.1. *Paclitaxel*

Paclitaxel, originally known as Taxol, a chemotherapeutic and a first-generation member of the taxane family, naturally occurring, originally isolated from the bark of the Pacific yew tree (Taxus brevifolia). Paclitaxel was discovered in the 1960s during a National Cancer Institute screening program aimed at identifying novel compounds with

anticancer potential. It showed strong anticancer activity but faced supply challenges due to unsustainable harvesting. This led to the development of semisynthetic production methods. After promising preclinical and clinical trials, paclitaxel was approved by the FDA in 1992 for ovarian cancer [173-175].

Paclitaxel delivers its antitumour effects primarily by binding to the β-subunit of tubulin, promoting microtubule polymerization and stabilization, which disrupts normal mitotic spindle formation, leading to G2/M phase cell cycle arrest and apoptosis. Unlike traditional chemotherapeutics, it does not damage DNA but induces cancer cell death through intrinsic and extrinsic apoptotic pathways, generation of ROS, inhibition of the PI3K/Akt pathway via PTEN activation, and modulation of miRNAs such as miR-22 and miR-145. Paclitaxel also enhances antitumour immunity by inhibiting regulatory T cells and TAMs, and it may induce autophagy, pyroptosis, senescence, and ferroptosis in a context-dependent manner. Its efficacy is often improved in combination with platinum-based drugs, radiotherapy, or natural compounds like resveratrol and curcumin, which help overcome resistance and reduce toxicity [174,176,181].

Paclitaxel is an effective, widely used chemotherapeutic. It is a standard treatment in ovarian cancer, often used in combination with agents like carboplatin or anthracyclines to improve survival outcomes. Its benefit as both a first- and second-line agent in breast cancer, with higher response rates seen in more aggressive, hormone receptor-negative tumours. In NSLCC, paclitaxel is part of combination regimens that enhance treatment response. It is also effective in Kaposi's sarcoma, particularly in immunocompromised patients, as well as in endometrial, bladder, and cervical cancers [177,178]. Despite its therapeutic success, paclitaxel is associated with adverse effects such as peripheral neuropathy, myelosuppression, and hypersensitivity reactions [179,180].

S1.1.8.2. Docetaxel

Docetaxel is a second-generation, semi-synthetic taxane derived from 10-deacetylbaccatin III extracted from the needles of the European yew tree and belongs to the same family as paclitaxel. Docetaxel was first approved in 1996 for the treatment of anthracycline-refractory metastatic breast cancer and subsequently for NSCLC, prostate cancer, and other solid tumours. Compared with paclitaxel, docetaxel demonstrates higher tumour cell accumulation, longer intracellular residence time, and greater in vitro cytotoxicity, with tumour cell reduction rates reported to be up to three times higher. These properties, combined with a lower incidence of hypersensitivity reactions, neurotoxicity, and hematologic toxicity, established docetaxel as a more potent and clinically versatile taxane [182,183].

Like paclitaxel, docetaxel exerts its antitumour effect by binding to the β-subunit of tubulin, promoting microtubule assembly and stabilizing these polymers against depolymerization. This hyperstabilization disrupts normal microtubule dynamics, blocking mitosis and leading to cell-cycle arrest and apoptosis. However, structural modifications at the 3′ and 10′ positions on the baccatin ring distinguish docetaxel from paclitaxel, leading to differences in potency, pharmacokinetics, and clinical activity. Docetaxel binds to microtubules with roughly twice the affinity of paclitaxel and exhibits stronger promotion of tubulin assembly, explaining its greater potency. Beyond mitotic arrest, docetaxel also triggers apoptosis through phosphorylation of the Bcl-2 protein, a process in which it is 10-100 times more potent than paclitaxel. Additionally, docetaxel shows significant anti-angiogenic activity, approximately four times stronger than paclitaxel and can inhibit vascular endothelial growth factor (VEGF)-mediated signaling. These multifaceted actions underpin its superior anti-tumour activity and provide a rationale for its use in combination with novel targeted agents [181-183,185,188].

Docetaxel is widely used as monotherapy or in combination regimens for a variety of solid tumours, including, head and neck; ovarian; anthracycline-refractory metastatic breast cancer [190]; NSCLC as a standard treatment option, frequently used in combination with platinum-based compounds; metastatic prostate cancer, both hormone-sensitive as well as resistant, with survival benefits observed in multiple phase III studies [186,189]; as well as in advanced gastric cancers for HER-2 negative patients [111]. Compared to paclitaxel, docetaxel has shown superior outcomes in certain settings, such as metastatic breast cancer, where it achieved longer time-to-progression and improved overall survival. Docetaxel's pharmacokinetic advantages, including linear kinetics and prolonged intracellular retention, contribute to its clinical efficacy. Moreover, its reduced risk of hypersensitivity reactions and neurotoxicity allows for broader use and more tolerable treatment schedules compared to paclitaxel [183,188].

S1.1.9. Rarely Used Agents

In addition to the commonly used chemotherapy drugs, several other anticancer agents see only sporadic use in PSCC. These include - pirarubicin, a 4'-O-substituted anthracycline analogue of doxorubicin that exhibits similar antitumour activity but with reduced cardiotoxicity. It is incorporated into tumour cells, where it inhibits DNA polymerase α and DNA synthesis [193]; peplomycin, a bleomycin analogue, which induces DNA strand breaks through microsome-catalysed oxidative mechanisms and subsequent binding to DNA. It shows pulmonary toxicity comparable to that of bleomycin [194]; actinomycin D, a DNA-intercalating antibiotic which binds to double-stranded DNA, forming reversible complexes. This blocks RNA transcription, leading to inhibition of protein synthesis and apoptosis [195]; vinorelbine, a vinca alkaloid like others binds to tubulin, disrupting microtubule assembly and causing G2/M phase arrest and apoptosis. It differs from vinca alkaloids described earlier by its higher selectivity for mitotic microtubules and lower neurotoxicity [162]; capecitabine - an oral prodrug that is converted to its only active metabolite, 5-FU, primarily within tumour tissue by thymidine phosphorylase, resulting in higher intratumoural 5-FU concentrations and reduced systemic exposure [197]. Other rarely used agents include irinotecan, a camptothecin derivative which inhibits topoisomerase I causing replication-dependent double-strand breaks, p53 activation, G2/M arrest, and apoptosis [196], similarly to topoisomerase II inhibitor - Etoposide [192]. Moreover, irinotecan also directly binds and inhibits MDM2 and Bcl-xL, enhancing p53 stabilization and mitochondrial-mediated apoptosis [196]; cabazitaxel a semisynthetic taxane binds to tubulin and stabilizes microtubules, leading to inhibition of mitosis. Unlike other taxanes, it does not have affinity for P-glycoprotein, allowing it to retain activity in tumours that are resistant to docetaxel [181]; finally, oxaliplatin, the third-generation platinum drug, like other platinum-based drugs, forms DNA crosslinks that block DNA replication and transcription, leading to apoptosis. What makes oxaliplatin unique is its bulkier DACH ligand, which underlying the properties of oxaliplatin-DNA adducts that are not recognized by MMR proteins, allowing the drug to remain effective in tumours resistant to cisplatin and carboplatin [75].

Although these agents are rarely used, their mechanisms of action align with those of their more established counterparts. Revisiting these compounds may yield valuable insights into the evolution of chemotherapeutic design and could inspire renewed research into why some were abandoned while others became mainstays of therapy in PSCC.
